# S-nitrosoglutathione inhibits adipogenesis in 3T3-L1 preadipocytes by S-nitrosation of CCAAT/enhancer-binding protein β

**DOI:** 10.1038/s41598-019-51579-x

**Published:** 2019-10-28

**Authors:** Marion Mussbacher, Heike Stessel, Teresa Pirker, Antonius C. F. Gorren, Bernd Mayer, Astrid Schrammel

**Affiliations:** 1 Department of Pharmacology and Toxicology, University of Graz, Humboldtstraße 46, A-8010 Graz, Austria; 2 Center for Physiology and Pharmacology, Department of Vascular Biology and Thrombosis Research, Medical University of Vienna, Schwarzspanierstraβe 17, A-1090 Vienna, Austria

**Keywords:** Nitrosylation, Obesity

## Abstract

Murine 3T3-L1 adipocytes share many similarities with primary fat cells and represent a reliable *in vitro* model of adipogenesis. The aim of this study was to probe the effect of S-nitrosoglutathione (GSNO) on adipocyte differentiation. Adipogenesis was induced with a mixture of insulin, dexamethasone, and 3-isobutyl-1-methylxanthine in the absence and presence of increasing GSNO concentrations. Biochemical analysis after 7 days of differentiation showed a prominent anti-adipogenic effect of GSNO which was evident as reduced cellular triglycerides and total protein content as well as decreased mRNA and protein expression of late transcription factors (e.g. peroxisome proliferator activated receptor γ) and markers of terminal differentiation (e.g. leptin). By contrast, the nitrosothiol did not affect mRNA and protein expression of CCAAT/enhancer-binding protein β (C/EBPβ), which represents a pivotal early transcription factor of the adipogenic cascade. Differentiation was also inhibited by the NO donor (Z)-1-[2-(2-aminoethyl)-N-(2-ammonioethyl)amino]diazen-1-ium-1,2-diolate. Biotin switch experiments showed significantly increased S-nitrosation of C/EBPβ variants indicating that posttranslational S-nitrosative modification of this transcription factor accounts for the observed anti-adipogenic effect of NO. Our results suggest that S-nitrosation might represent an important physiological regulatory mechanism of fat cell maturation.

## Introduction

As an important signaling molecule NO mediates a broad range of physiological functions including control of vessel tone and platelet aggregation, neuronal communication, and immune response (for review, see^1^). Furthermore, NO seems involved in cellular processes such as proliferation, differentiation, and apoptosis. NO bioactivity has been described to be mainly mediated by binding to protoporphyrin-IX-type heme containing soluble guanylyl cyclase, accumulation of cGMP, and consequent activation of protein kinase G (for reviews, see^2,3^). However, NO can also act in a direct manner independently of guanylyl cyclases, triggering S-nitrosation of one or multiple cysteine residues in proteins, leading to changes in activity, stability, protein-protein interactions, and/or localization of the respective targets. A huge number of *S*-nitrosated proteins has been identified in the last years in physiological and pathological settings e.g. hemoglobin^4^, *N*-methyl-D-aspartate receptor^5^, caspase-3^6^, and I-κB kinase^7^ and S-nitrosation is commonly regarded as ubiquitous posttranslational protein modification.

Adipocyte differentiation *i.e*. the development of mature fat cells from precursors in response to adipogenic stimuli is a complex and tightly regulated process that is characterized by distinct phases and key transcription factors. The immortalized preadipocyte 3T3-L1 cell line^8^ represents a well-characterized and widely used cell model to study diverse aspects of fat cell biology. When preadipocytes reach a confluent state, cells temporarily enter a stage of growth arrest due to contact inhibition. Upon experimental challenge with mitotic and adipogenic inducers (insulin, glucocorticoids, cAMP elevating compounds, and growth hormones) cells synchronously re-enter the G_1_ phase of the cell cycle and undergo several rounds of mitosis. This so-called mitotic clonal expansion is driven by expression of oncogenes *c-jun*, *c-fos*, *c-myc* as well as early transcription factor CCAAT/enhancer-binding protein subtypes β and δ (C/EBPβ and C/EBPδ)^9^. These transiently expressed proteins are known to play a pivotal role in the induction of late adipogenic factors *i.e*. peroxisome proliferator activated receptor γ (PPARγ), C/EBPα, and sterol regulatory element binding protein 1 (SREBP-1). Activation of PPARγ induces expression of C/EBPα which acts anti-mitotically on the one hand and stimulates PPARγ protein biosynthesis on the other hand^10^. *Via* this positive feedback loop adipocytes generate high levels of PPARγ and C/EBPα, which synergistically promote the terminal phase of adipogenesis that is characterized by expression of a broad range of proteins that are required for maintenance of the mature adipogenic phenotype (e.g. lipogenic and lipolytic enzymes, fatty acid binding proteins, and leptin). Moreover, additional positive feedback loops from PPARγ back to C/EBPβ and eventually to the insulin receptor have been reported^11^. This complex cooperative network guarantees irreversible transition of preadipocytes into mature adipocytes.

Recently, compelling evidence indicated that S-nitrosation of key transcription factors is an important process controlling adipogenesis. In particular, PPARγ was found to be sensitive to S-nitrosative modification^12,13^. The aim of the present study was to investigate the effect of S-nitrosation on the adipogenic cascade and to identify additional targets of S-nitrosation in greater detail by probing the effect of S-nitrosoglutathione (GSNO) on differentiation of 3T3-L1 cells.

## Results

### Effects of GSNO and DETA/NO on differentiation of 3T3-L1 cells

**Figure 1:**
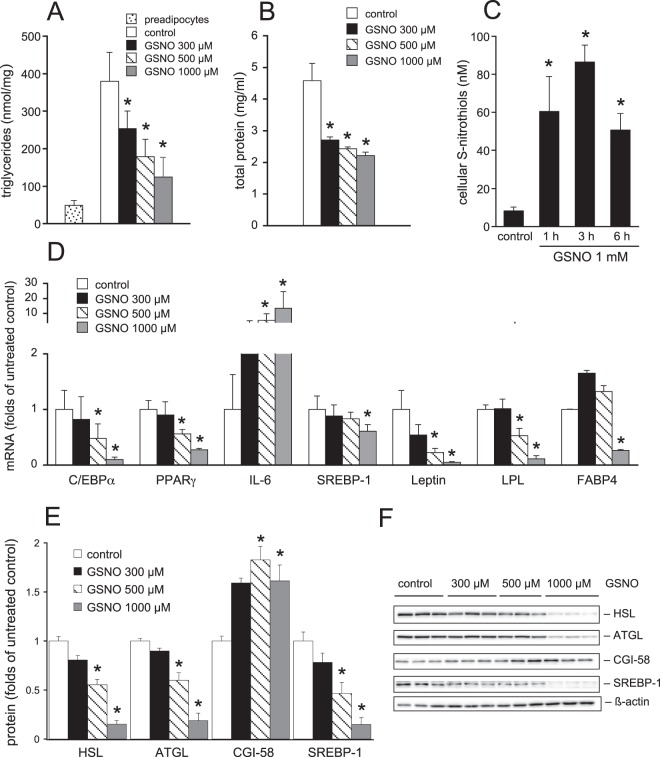
Effect of GSNO on adipogenesis of 3T3-L1 cells (protocol A). Formation of cellular TGs (**A**) and protein (**B**) was reduced by GSNO in a concentration-dependent manner. Intracellular formation of S-nitrosothiols in response to exogenous GSNO. (**C**) mRNA expression of C/EBPα, PPARγ, SREBP-1, leptin, LPL, and FABP4 was downregulated in the presence of GSNO whereas IL-6 mRNA expression was significantly increased. (**D**) In whole-cell lysates, protein levels of SREBP-1, ATGL, and HSL were reduced, while CGI-58 protein was increased in the presence of GSNO. (**E**) Representative Western Blots. (**F**) Data represent mean values ± SEM of 3 individual experiments; *p < 0.05 *vs* untreated control.

To investigate the effect of GSNO on adipogenesis, 3T3-L1 cells were differentiated for 7 days in the absence and presence of increasing concentrations of the thionitrite GSNO (protocol A). As illustrated in Fig. 1A, formation of triglycerides (TGs) was significantly inhibited by GSNO (300 µM–1 mM) in a concentration-dependent manner, indicating decreased adipocyte differentiation. This effect was associated with reduced cellular protein content in the presence of the S-nitrosothiol (Fig. 1B). Since inhibition of adipogenesis by GSNO was observed at rather high concentrations of the thionitrite (>100 µM), we wanted to estimate the concentration of S-nitrothiols within the cell. Therefore, 3T3-L1 cells were incubated with GSNO (1 mM) and the endogenous formation of S-nitrosothiols was quantified as HgCl_2_-sensitive production of nitrite. Surprisingly, we found that the concentration was in the range between ~60–90 nM (Fig. 1C). Thus, less than 0.01% of GSNO-derived NO was converted into intracellular high (protein) or low molecular weight thionitrites in our experiments^14^. Extrapolation of the data shown in Fig. 1A (with IC_50_ values for GSNO in the range of 300–500 µM) suggests an intracellular IC_50_ for GSNO between 20 and 50 nM, which would be clearly in the physiological range^15^. Real-time quantitative PCR experiments revealed significantly decreased mRNA levels of C/EBPα, PPARγ, and SREBP-1, which are well-known key players of the adipogenic process (Fig. 1D). Furthermore, leptin, lipoprotein lipase (LPL), and fatty acid-binding protein 4 (FABP4) that are expressed in the terminal phase of adipogenesis were massively downregulated in GSNO-treated cells. Interestingly, mRNA levels of the proinflammatory cytokine interleukin 6 (IL-6), which was reported to be higher in preadipocytes as compared to differentiated adipocytes^16^, were upregulated in a concentration-dependent manner: At the highest GSNO concentration tested, a ~10-fold increase of IL-6 mRNA was observed compared to untreated cells. To verify our results on protein expression levels, we performed Western Blot analysis of the transcription factor SREBP-1 and of (co)lipases adipose triglyceride lipase (ATGL), hormone-sensitive lipase (HSL), and comparative gene identification-58 (CGI-58) as illustrated in Fig. 1E,F. While protein levels of SREBP-1, ATGL, and HSL were decreased upon treatment of cells with the thionitrite, cellular CGI-58 expression was increased in the presence of increasing GSNO concentrations.

**Figure 2:**
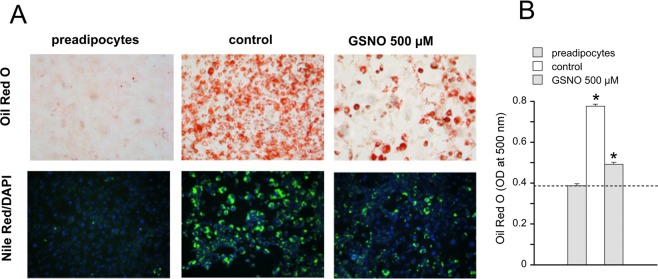
Effect of GSNO (500 µM) on formation of TGs was visualized by staining of cells with Oil Red O (red) or Nile Red (green). DAPI (blue) was used as nuclear counterstain. (**A**) Spectroscopic quantification of eluted Oil Red O dye. (**B**) Data represent mean values ± SEM of 4 individual experiments. *p < 0.05 *vs* preadipocytes.

**Figure 3:**
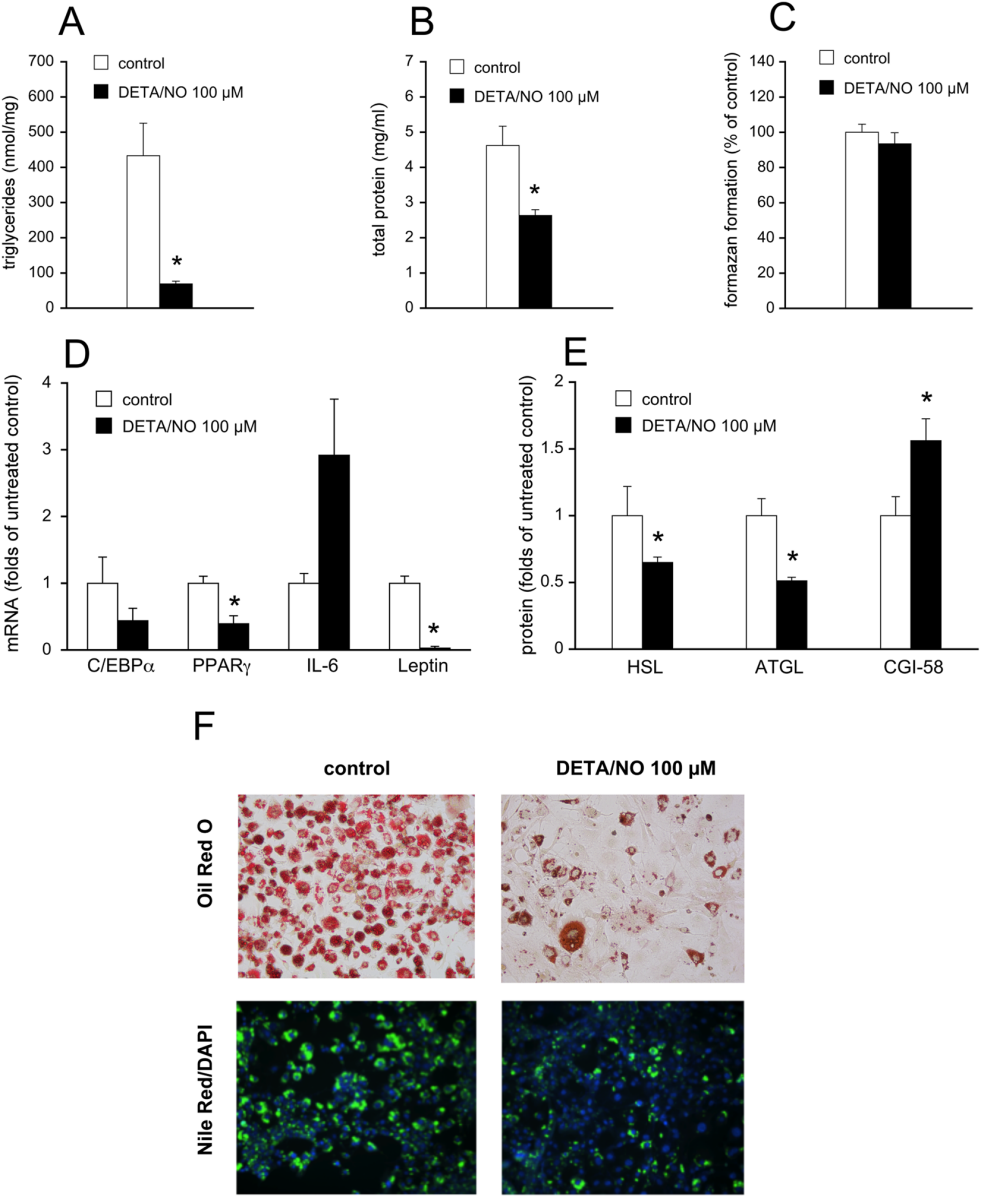
Effect of DETA/NO on adipogenesis of 3T3-L1 cells (protocol A). Formation of cellular TGs (**A**) and protein (**B**) was reduced by treatment with DETA/NO (100 µM). Cell viability was not affected by DETA/NO. (**C**) mRNA expression of PPARγ and leptin (D) as well as protein expression of HSL and ATGL (**E**) were significantly downregulated by DETA/NO. By contrast, protein levels of CGI-58 were increased by treatment with the NO donor. (**E**) Protein expression was analyzed in whole-cell lysates. Data represent mean values ± SEM of 3 individual experiments; *p < 0.05 *vs* untreated control. Effect of DETA/NO on formation of TGs was visualized by staining with Oil Red O and Nile Red/DAPI (**F**).

To exclude a cytotoxic effect of GSNO on 3T3-L1 adipocytes, cell viability was measured using the 3-(4,5-di-methylthiazol-2-yl)-2,5-diphenyltetrazoliumbromide (MTT) test. We detected a slight reduction of formazan formation (–20%) in the presence of GSNO concentrations ≥500 µM (Supplemental Fig. S1A), which could be explained by decreased cell numbers observed with GSNO-treated cells. Nevertheless, we measured activation of caspase-3 monitored as cleavage of the full-length enzyme (MW ∼35 kDa) into a shorter form (MW ∼17/19 kDa) as a second approach to judge potential cell death (apoptosis). Ratios of cleaved caspase to total caspase were similar between untreated and GSNO-treated cells (Supplemental Fig. S1B). Moreover, cleavage of poly (ADP-ribose)-polymerase (PARP; *i.e*. one of the main targets of caspase-3) was not observed in GSNO-treated cells indicating that the compound does not induce apoptosis (Supplemental Fig. S1C). To visualize the effect of GNSO on adipocyte differentiation, intracellular lipids were stained in preadipocytes as well as in untreated and GSNO-treated adipocytes with Oil Red and Nile Red (Fig. 2A). Accumulation of TGs was significantly blunted in GSNO-treated cells compared to controls, whereas lipids were hardly detectable in preadipocytes. In Fig. 2B, the spectroscopic quantification of the eluted Oil Red O dye is illustrated. To probe if the observed effect on adipogenesis is limited to S-nitrosothiols some key experiments were performed with (Z)-1-[2-(2-aminoethyl)-N-(2-ammonioethyl)amino]-diazen-1-ium-1,2-diolate (DETA/NO), a compound that releases free NO radical with a half-life of 20 h (37 °C)^17^. As shown in Fig. 3, DETA/NO (100 µM) induced similar or even more pronounced effects on cellular TG and protein levels (Fig. 3A,B), on mRNA expression of PPARγ and leptin (Fig. 3D) as well as on protein expression of ATGL, HSL, and CGI-58 (Fig. 3E). As evident from the MTT test, the NO donor did not affect cell viability at the applied concentration (Fig. 3C). Moreover, Oil Red and Nile Red stainings of DETA/NO-treated cells exhibited a staining pattern similar to that of GSNO-treated cells (Fig. 3F**)**.

### Short- and long-term actions of GSNO

**Figure 4:**
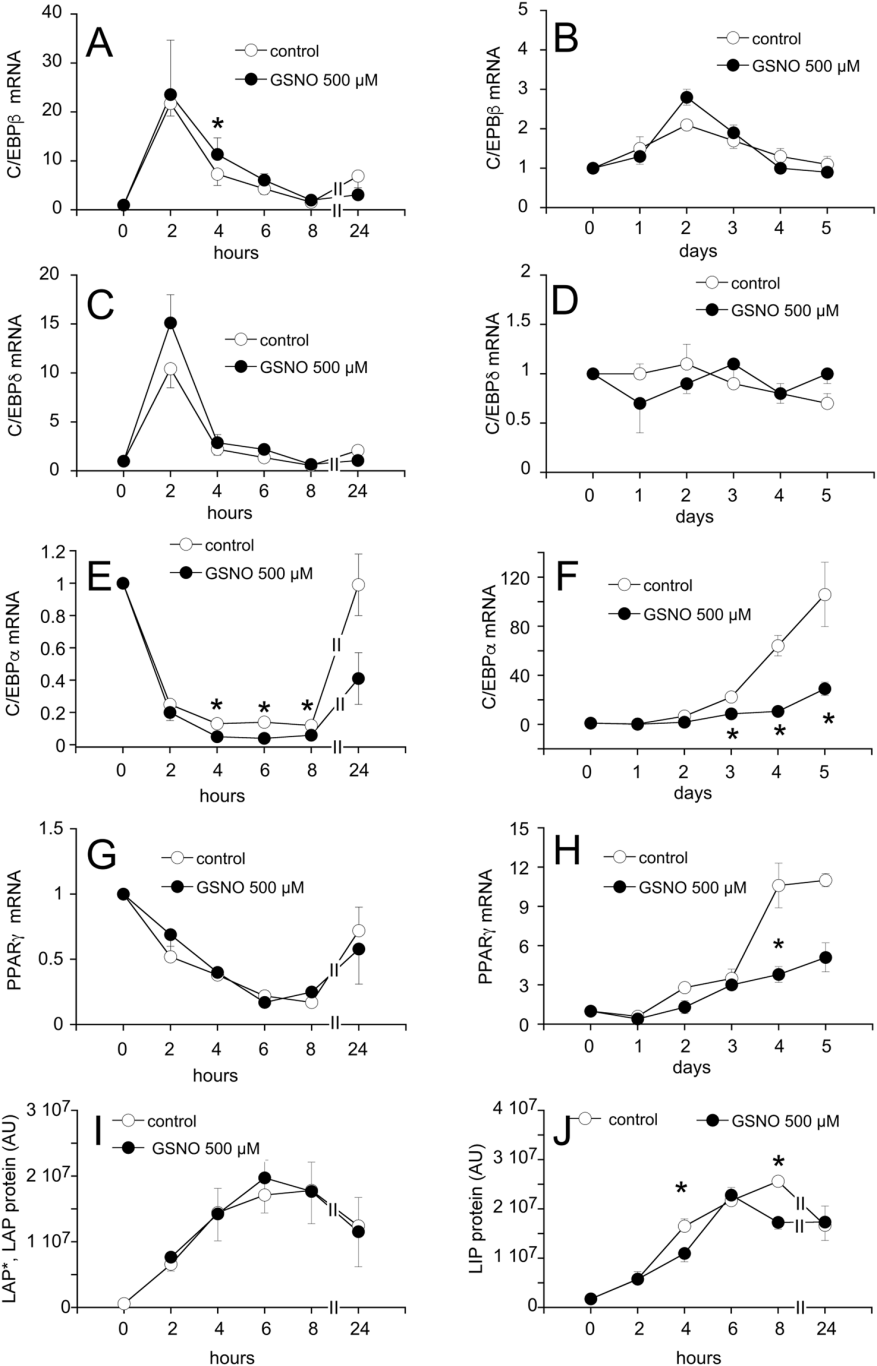
Effect of GSNO (500 µM) on mRNA and protein expression of transcription factors. Long- and short-term experiments were performed according to protocols B and C, respectively. GSNO treatment neither affected C/EBPβ mRNA levels (**A,B**) nor protein expression of LAP*, LAP, and LIP isoforms (**I,J**). Time course of C/EBPδ mRNA expression was similar in the absence and presence of GSNO (**C,D**). GSNO significantly decreased C/EBPα mRNA levels in the initial phase of adipogenesis (**E**; 4–8 h after induction). Downregulation of C/EBPα mRNA expression persisted in the late phase of adipogenesis (**F**). PPARγ mRNA levels were not affected by GSNO over the first 24 h *post* induction (**G**). Inhibition of PPARγ mRNA expression became evident on day 4 of differentiation and persisted in the late phase of adipogenesis. (**H**) Cellular mRNA levels are expressed as folds of untreated control and represent mean values ± SEM of 3 individual experiments; *p < 0.05 *vs* untreated control.

Differentiation of 3T3-L1 cells is characterized by a series of temporally coordinated steps carried out by distinct transcription factors of the adipogenic machinery. To identify potential targets of GSNO, different sets of time course experiments were performed. First, preadipocytes were incubated in differentiation media up to 24 h in the absence and presence of GSNO (500 µM). Cells were harvested at 2, 4, 6, 8, and 24 h and analyzed for mRNA and protein expression of various transcription factors (protocol C). In another, long-term approach, adipogenesis was followed for 5 days (protocol B) and cells were harvested daily and analyzed accordingly. As shown in Fig. 4, mRNA levels of C/EBPβ (Fig. 4A) and C/EBPδ (Fig. 4C) peaked at 2 h, whereas mRNA levels of C/EBPα (Fig. 4E), and PPARγ (Fig. 4G) declined in the short-term incubation set-up starting at the 2 h time point. Interestingly, C/EBPα mRNA was downregulated ~2–3-fold upon challenge with GSNO, an effect that became evident 4 h after induction of adipogenesis (Fig. 4E). By contrast, mRNA levels of C/EBPβ were significantly increased at this time point, indicating that C/EBPβ and C/EBPα might play an important early role in mediating the effect of GSNO on adipogenesis. Interestingly, protein expression of C/EBPβ isoforms liver-enriched activator protein (LAP) and liver-enriched activator protein* (LAP*; Fig. 4I) was not affected within this time frame, however, the liver-enriched inhibitory protein (LIP; Fig. 4J) was significantly decreased after 4 and 8 h. Interestingly, analysis of long-term experiments did not reveal significant effects of GSNO on C/EBPβ (Fig. 4B) and C/EBPδ (Fig. 4D) mRNA levels, but showed a prominent and time-dependent decrease of C/EBPα and PPARγ mRNA expression compared to untreated controls, indicating that GSNO acts on a transcription factor regulating C/EBPα and PPARγ expression. Results are shown in Fig. 4F,H, respectively.

**Figure 5:**
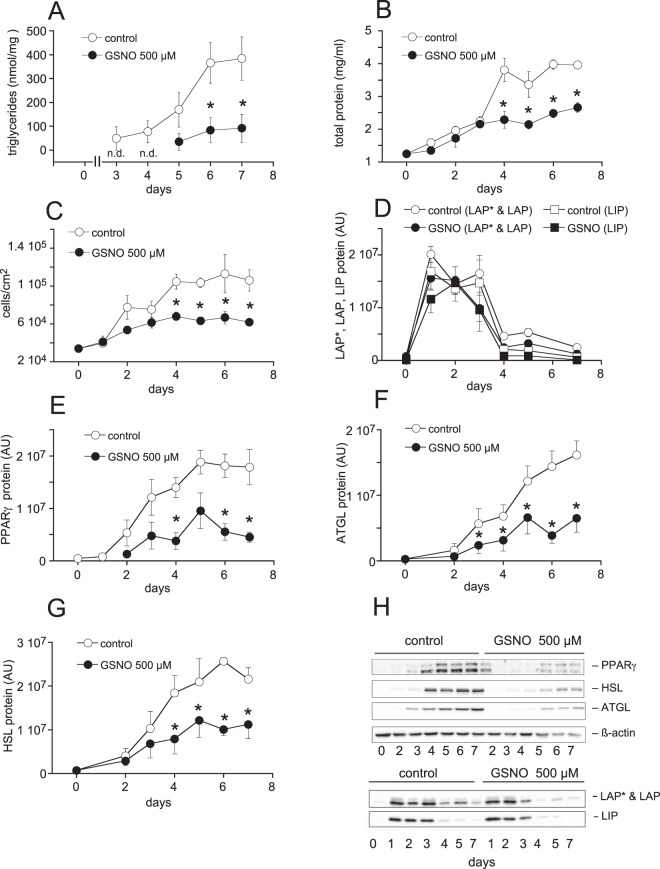
Effect of GSNO on time-dependent cell proliferation and adipogenesis. GSNO (500 µM) inhibited formation of TGs (**A**), cellular protein levels (**B**) and cell proliferation (**C**) in a time-dependent manner. Protein expression of C/EBPβ isoforms in whole-cell lysates was not affected (**D**). PPARγ (**E**), ATGL (**F**), and HSL (**G**) protein levels were significantly downregulated in the presence of GSNO. (**H**) Representative Western blots. Data represent mean values ± SEM of 3–4 individual experiments; *p < 0.05 *vs* untreated control.

In a next step, we monitored triglyceride content, total protein, cell number as well as protein expression of important lipogenesis-associated proteins over a period of 7 days (protocol B). Adipogenesis was delayed and attenuated in the presence of GSNO (500 µM) as evident from decreased cellular TG levels over the whole period of the experiment (Fig. 5A). By contrast, total protein content (Fig. 5B) and cell number (Fig. 5C) were not affected by the thionitrite until day 3 of differentiation. Subsequently, while control cells continued to proliferate, GSNO-treated cells did not significantly propagate until day 7, indicating both anti-adipogenic and anti-proliferative effects of the S-nitrosothiol. While protein expression of C/EBPβ isoforms LAP* (38 kDa), LAP (35 kDa), and LIP (21 kDa) was not affected by GSNO treatment (Fig. 5D), we found that expression of PPARγ, ATGL, and HSL was significantly reduced (Fig. 5E–G), further supporting our data obtained with qPCR. In Fig. 5H representative Western blots are shown.

### Insulin- and PPARγ-independent inhibition of adipogenesis

**Figure 6:**
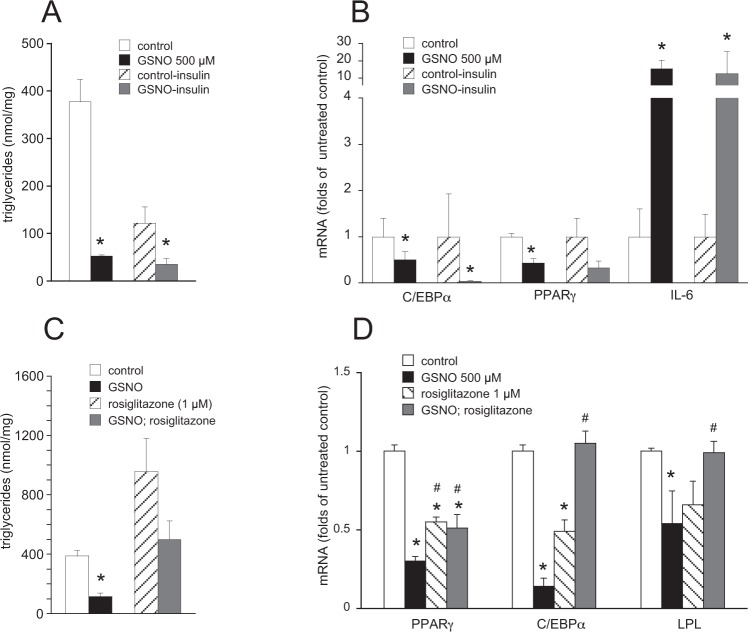
Insulin- and PPARγ-independent inhibition of adipogenesis. Omission of insulin from the induction cocktail neither influenced the inhibitory effect of GSNO on formation of TGs (**A**) nor on mRNA expression of C/EBPα cocktail for 7 days neither and IL-6 (**B**); *p < 0.05 *vs* untreated control. Co-treatment of 3T3-L1 cells with GSNO (500 µM) and the PPARγ agonist rosiglitazone (1 µM) restored cellular TGs to at least control levels (**C**) and completely reversed the effect of GSNO on formation of C/EBPα and LPL mRNA expression (**D**). Data represent mean values ± SEM of 3 individual experiments; *p < 0.05 *vs* untreated control; # p < 0.05 *vs* treatment with GSNO (500 µM).

To shed light on the mechanism underlying the effect of GSNO we probed the hypothesis that the S-nitrosothiol might interfere with the action of insulin, which represents a potent inducer of adipogenesis. Cells were differentiated in the absence and presence of insulin with and without GSNO (500 µM) and cellular TGs as well as mRNA levels of C/EBPα, PPARγ, and IL-6 were analyzed. As illustrated in Fig. 6A, omission of insulin from the induction medium led to a prominent reduction of cellular TG formation under control conditions. Under insulin-deficient conditions, TG levels were decreased from 121 ± 22 to 35 ± 13 nmol × mg^-1^ in the absence and presence of GSNO (500 µM), respectively, indicating that insulin-independent adipogenesis is also sensitive to the S-nitrosothiol. Similarly, the effect of GSNO on C/EBPα, and IL-6 mRNA expression persisted in the absence of insulin (Fig. 6B). Albeit data did not reach statistical significance for PPARγ mRNA expression under insulin-deficient conditions (Fig. 6B) our results largely exclude interference of the thionitrite with insulin-dependent adipogenesis as active principle. Recently, S-nitrosation of PPARγ by inducible nitric oxide synthase (iNOS)-derived NO has been described to negatively regulate stability and activity of the transcription factor^13^. To evaluate if PPARγ signaling is functionally impaired by GSNO in our experimental approach, cells were co-incubated with the synthetic PPARγ agonist rosiglitazone (1 µM) for 7 days (protocol A). Treatment of control cells with the thiazolidinedione led to a ~2.5-fold increase in TG levels, confirming the adipogenic properties of the drug (Fig. 6C). GSNO-treated cells were also sensitive to the PPARγ agonist since cellular TG levels were increased from 117 ± 23 to 500 ± 126 nmol × mg^−1^ in the absence and presence of rosiglitazone, respectively. Thus, activation of PPARγ by an exogenous agonist in the presence of GSNO yielded TG values comparable to or even higher than those of untreated control cells (390 ± 36 nmol × mg^−1^). Similarly, the drug completely restored mRNA levels of C/EBPα and LPL in the presence of GSNO (Fig. 6D), indicating that rosiglitazone-activated PPARγ signaling is barely affected by the thionitrite.

### Time-dependent action of GSNO

**Figure 7:**
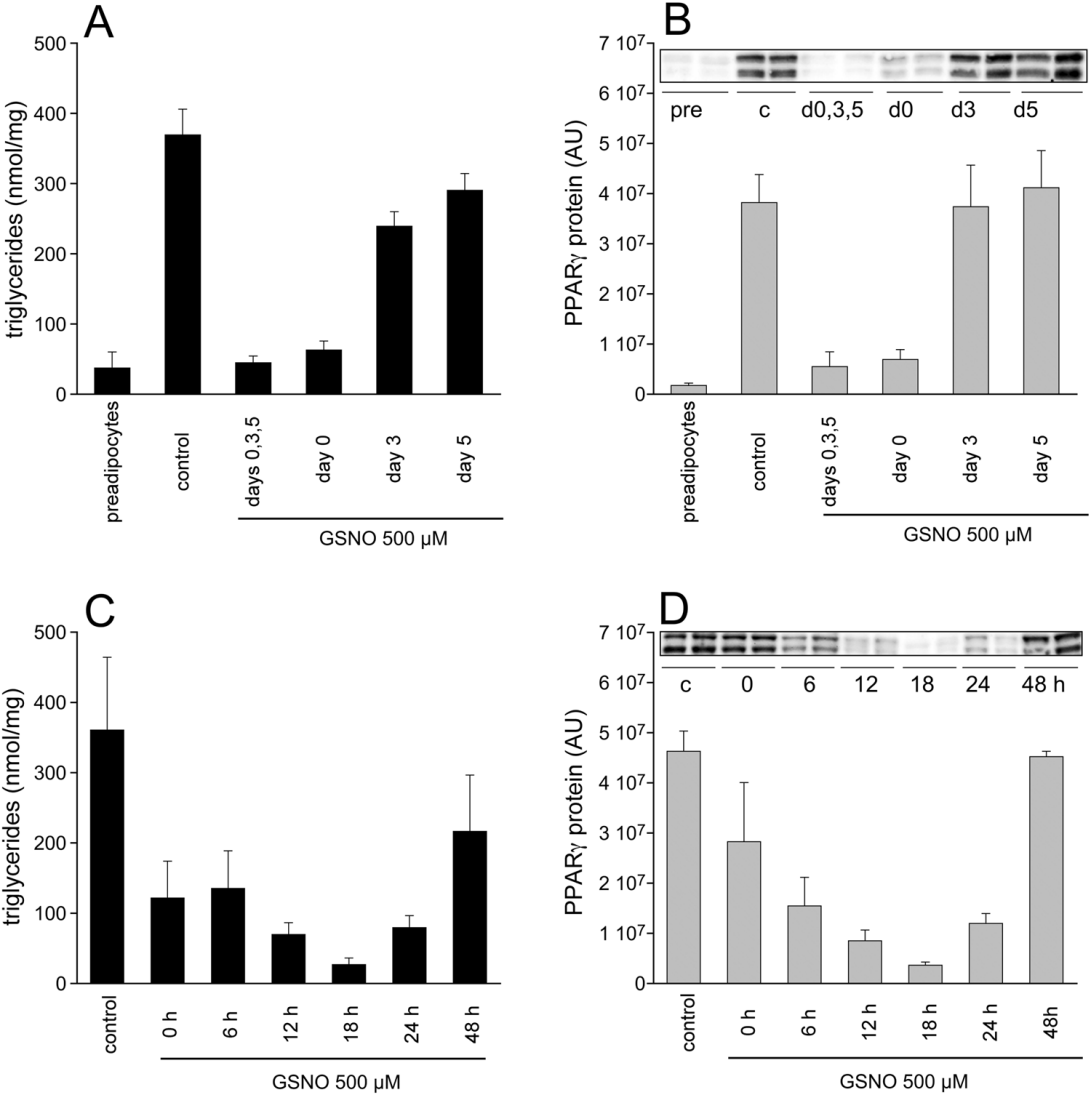
Time-dependent effects of GSNO. Formation of TGs (**A**) and PPARγ protein expression (**B**) analyzed 7 days after induction of adipogenesis was most affected when GSNO (500 µM) was applied according to protocol A or *via* single treatment of cells at day 0. Application of GSNO 18 h after start of differentiation exerted the most prominent effect on formation of TGs (**C**) and protein expression of PPARγ (**D**). Protein expression was analyzed in whole-cell lysates. Data represent mean values ± SEM of 4 individual experiments; pre (preadipocytes); c (control); d0,3,5 (application of GSNO at days 0, 3, and 5 of differentiation); d0 (application at day 0); d3 (application at day 3); d5 (application at day 5).

Differentiation of 3T3-L1 cells is routinely performed according to a standard protocol that comprises sequential adipogenic stimuli (protocol A). Thus, GSNO was repeatedly applied in most of our experiments. To investigate if a single application is sufficient or whether a repeated challenge is necessary to mediate the observed effects, cells were treated with GSNO (500 µM) at different time points and with different frequencies. After 7 days of differentiation, cellular TGs and PPARγ protein were analyzed. As illustrated in Fig. 7A, a single application of GSNO (500 µM) at day 0 of differentiation was sufficient to exert prominent inhibition of adipogenesis. Treatment of cells in advanced states of differentiation resulted in attenuation (day 3) or absence (day 5) of inhibition. The profile of PPARγ protein expression under these conditions was in excellent accordance with that of TG formation (Fig. 7B). To characterize the early stage of differentiation in more detail, effects of single GSNO applications at 0, 6, 12, 18, 24, or 48 h after induction were tested accordingly (Fig. 7C,D). Cells were highly sensitive to application of GSNO 18 h after start of differentiation, indicating that the thionitrite interacts with an adipogenic factor that is maximally active within this time frame.

### S-nitrosation of C/EBPβ

**Figure 8:**
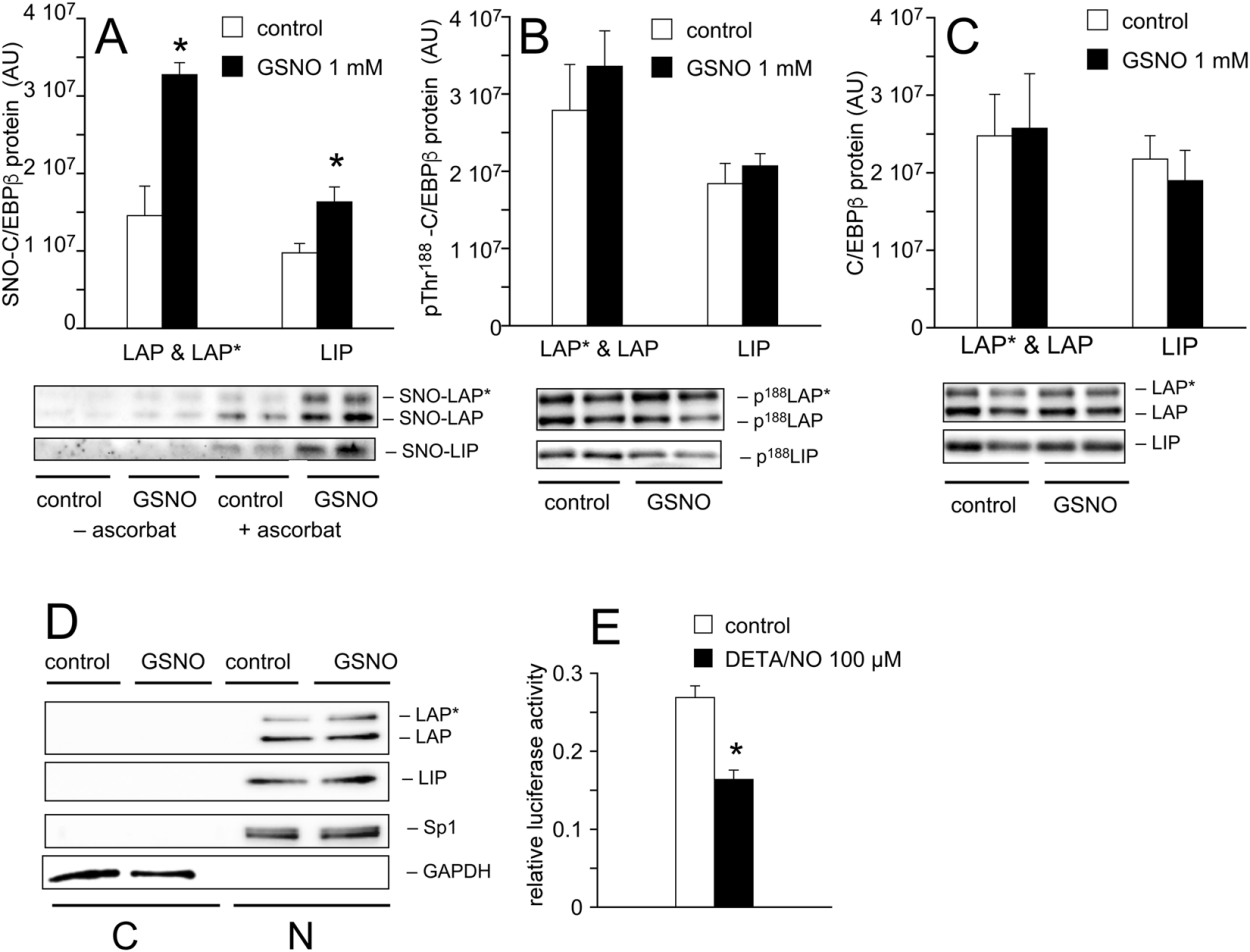
S-nitrosation of C/EBPβ impairs transcriptional activity. Differentiation of 3T3-L1 cells in the presence of GSNO (1 mM) for 6 h resulted in significant S-nitrosation of LAP*, LAP, and LIP in crude nuclear preparations. (**A**) Phosphorylation of C/EBPβ at Thr188 (**B**) as well as nuclear levels of C/EBPβ protein (**C**) were not affected by GSNO. Data represent mean values ± SEM of 5 individual experiments; *p < 0.05 *vs* untreated control. Cytosolic and nuclear-enriched fractions were characterized by Western blot using GAPDH and the transcriptional factor Sp1 as marker proteins, respectively (**D**). C/EBPβ proteins LAP*, LAP, and LIP were enriched in the nuclear fraction; C (cytosolic fraction), N (nuclear fraction). (**E**) Transcriptional activity of C/EBPβ was measured in HEK 293 cells using a dual luciferase reporter assay. Relative luciferase activity was significantly decreased in the presence of DETA/NO (100 µM). Data represent mean values ± SEM of 8 individual experiments; *p < 0.05 *vs* untreated control.

Analysis of mRNA and protein profiles of important transcription factors as well as determination of the time-dependent actions of GSNO revealed that the thionitrite acts at a very early time point of adipocyte differentiation (within 24 h). Since C/EBPβ mRNA expression peaked very early and was not affected by GSNO, we hypothesized that transcriptional activity of C/EBPβ might be affected by GSNO. Using the biotin switch method, S-nitrosation of C/EBPβ was studied in nuclear-enriched fractions prepared from untreated and GSNO-treated cells harvested 6 h *post* induction. As shown in Fig. 8A, S-nitrosation of C/EBPβ isoforms LAP*, LAP, and LIP was barely detectable in nuclear extracts of control cells. Differentiation of 3T3-L1 cells in the presence of GSNO (1 mM) yielded significantly increased S-nitrosation of the transcription factor. Compared to control preparations, S-nitrosation of LAP* and LAP as well as LIP was upregulated 2.8 ± 0.5 and 1.7 ± 0.1-fold, respectively. In contrast, phosphorylation of C/EBPβ variants at Thr^188^ (Fig. 8B) as well as nuclear expression of C/EBPβ isoforms (Fig. 8C) were not affected by GSNO. Quality of the nuclear preparation is shown in Fig. 8D using specificity protein 1 (Sp1) and glyceraldehyde-3-phosphate dehydrogenase (GAPDH) as nuclear and cytosolic markers, respectively. Time course experiments revealed that S-nitrosation of C/EBPβ was maximal at 6 h of differentiation, however, the effect persisted at least for 48 h (Supplemental Fig. S2A,C). As a final step, we investigated whether increased S-nitrosation affects transcriptional activity of C/EBPβ. Therefore, a dual luciferase reporter assay was performed in human embryonic kidney 293 (HEK 293) cells. Co-transfection of cells with a plasmid encoding C/EBPβ with a firefly luciferase construct under the control of a C/EBP response element for PPARγ was performed in the absence and presence of DETA/NO (100 μM) for 48 h. As illustrated in Fig. 8E, presence of the NO donor significantly decreased C/EBPβ-dependent luciferase activity by ~40%. A similar reduction of luciferase activity was observed when co-transfected cells were challenged with GSNO (500 μM) every 8 h (data not shown), indicating impaired activity of C/EBPβ as the underlying mechanism for decreased adipogenesis in the presence of GSNO.

## Discussion

**Figure 9:**
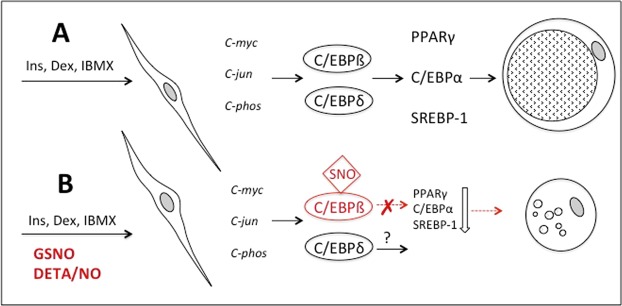
Proposed mechanism of NO-mediated inhibition of adipogenesis. Differentiation of 3T3-L1 cells was initiated with a cocktail of insulin (Ins), dexamethasone (Dex), and IBMX in the absence (**A**) and presence (**B**) of NO donors GSNO and DETA/NO. Inhibition of the early transcription factor C/EBPβ by NO-mediated S-nitrosation results in suppression of the adipogenic cascade. Expression of late adipogenic factors PPARγ, C/EBPα, SREBP-1 as well as of adipocyte proteins characteristic for terminal differentiation is severely blunted in the presence of GSNO resulting in an adipocyte phenotype with significantly reduced TG levels.

In this study we demonstrate for the first time that the early adipogenic transcription factor C/EBPβ is S-nitrosated by GSNO and suggest that this posttranslational modification is associated with reduced transcriptional activity and impaired adipogenesis. This hypothesis is corroborated by results showing that although C/EBPβ mRNA and protein levels are not affected by the thionitrite, subsequent downstream targets PPARγ, C/EBPα, and SREBP-1 are significantly downregulated. As a final consequence of the suppressed adipogenic cascade, expression of late proteins characterizing the mature adipocyte phenotype (e.g. ATGL, HSL, and LPL) is blunted or retarded (Fig. 9).

C/EBPβ was reported to undergo a series of posttranslational modifications including phosphorylation, acetylation, methylation, sumoylation, and ubiquitination (for reviews, see^18,19^). Sequential phosphorylation of C/EBPβ at distinct threonine and serine residues was associated with acquisition of DNA binding activity and stability of the transcription factor. In 3T3-L1 cells, maximal phosphorylation and transcriptional activity of C/EBPβ was reported to occur 16–24 h after induction of differentiation^20^. Interestingly, we did not observe any effect of GSNO on Thr^188^ phosphorylation of C/EBPβ, a modification that primes the transcription factor for subsequent phosphorylation. Instead, we could show that incubation of preadipocytes with GSNO leads to S-nitrosation of the C/EBPβ protein, which was associated with decreased transcriptional activity. Time course experiments revealed that a single application of GSNO exerted the most prominent inhibitory effect on adipogenesis after 18 h of differentiation, reflecting the peak of transcriptional activity of C/EBPβ and indicating that the thionitrite affects C/EBPβ protein in a very sensitive state.

Moreover, the effect of GSNO on fat cell maturation occurred independently of insulin signaling. Omission of the hormone from the induction cocktail resulted in attenuated adipogenesis but did not prevent the inhibitory effect of the S-nitrosothiol. These results indicate that a mechanism involving S-nitrosation of insulin-sensitive targets including insulin receptor β, insulin receptor sensitive substrate 1 or protein kinase B/Akt^21^ is not involved in the observed antiadipogenic effect. Moreover, no significant effect of GSNO on expression and/or activity of AMP-activated protein kinase (AMPK; Supplemental Fig. S3) was observed excluding a major contribution of the AMPK pathway to inhibition of adipogenesis by NO.

Thus far, it is not known which cysteine residue(s) of the C/EBPβ protein is/are modified by GSNO. Members of the C/EBP family are commonly composed of distinct structural components *i.e*. a highly conserved C-terminal leucine-zipper dimerization motif, a basic DNA binding region, a regulatory domain, and a transactivating region located at the N-terminus of the protein (for reviews, see^18,19^). Due to existence of alternative translation initiation sites within the C/EBPβ mRNA^22,23^ there exist three different protein variants with distinct biological functions, that is full-length C/EBPβ (LAP*; 38 kDa), LAP (35 kDa), and LIP (21 kDa). Within its primary structure, murine LAP*, LAP, and LIP contain six, five, and two cysteine residues, respectively. None of them is located within the conserved leucine-zipper motif. Western blot experiments performed with an antibody that detects these isoforms with different sensitivity suggest that all C/EBPβ proteins are significantly S-nitrosated. Regarding the LIP isoform, however, this result should be treated with caution, since blot signals of the S-nitrosated protein were near the detection limit and therefore hard to quantify. Site-directed mutagenesis will be necessary to ultimately identify the cysteine residue(s) affected by GSNO.

Recently, it was demonstrated that PPARγ is also sensitive to S-nitrosative modification. Cao *et al*. showed that treatment of PPARγ-transfected HEK 293 cells with GSNO resulted in increased S-nitrosation of the transcription factor that was accompanied by decreased transcriptional activity^12^. A similar effect of the S-nitrosothiol on transcriptional activity of PPARγ was observed in PPARγ-transfected HeLa cells^13^. In that study the S-nitrosation site of recombinant PPARγ was identified as Cys^168^ by mass spectrometry. Our results revealed that the transcription factor C/EBPβ is an additional target of S-nitrosation that contributes to decreased adipogenesis in the presence of GSNO. Moreover, our data indicate that C/EBPβ nitrosation is a very early event during adipocyte differentiation that affects subsequent physiological processes.

NOS isoforms are the endogenous enzymatic source of NO, however, their expression in preadipocytes is very low. In a physiological context it is most likely that NO is derived from neighbouring cells within white adipose tissue, which represents a specialized connective tissue with metabolic and endocrine functions and is composed of different cell types including not only preadipocytes and mature adipocytes, but also endothelial cells, neurons, macrophages, and other immune cells. Recently, it was demonstrated that co-culture of 3T3-L1 cells with lipopolysaccharide-activated RAW 264.7 macrophages resulted in S-nitrosation of PPARγ by iNOS-derived NO^13^. Thus, infiltrating macrophages or activation of tissue-resident macrophages in immediate proximity to fat cells represent likely candidates for production of supraphysiological amounts of NO *via* activation of iNOS. Thus, sequential S-nitrosative modification of transcription factors might represent an important mechanism to control fat cell maturation under inflammatory conditions *in vivo*.

## Materials and Methods

### Materials

3T3-L1 cells (ATCC^®^ CL-173™) were purchased from ATCC® *via* LGC Standards GmbH (Wesel, Germany). HEK 293 cells were kindly provided by Prof. Wolfgang Graier (Department of Molecular Biology and Biochemistry, Medical University Graz, Austria). GSNO and DETA/NO were obtained from Enzo Life Science (Lausen, Switzerland). Information about antibodies is provided in Supplemental Table 1. Complete Protease Inhibitor^TM^ Cocktail and PhosSTOP^™^ Phosphatase Inhibitor Cocktail were from Roche Life Science (Vienna, Austria). All other chemicals (unless otherwise indicated) were obtained from Sigma (Vienna, Austria).

### Cell culture

**Figure 10:**
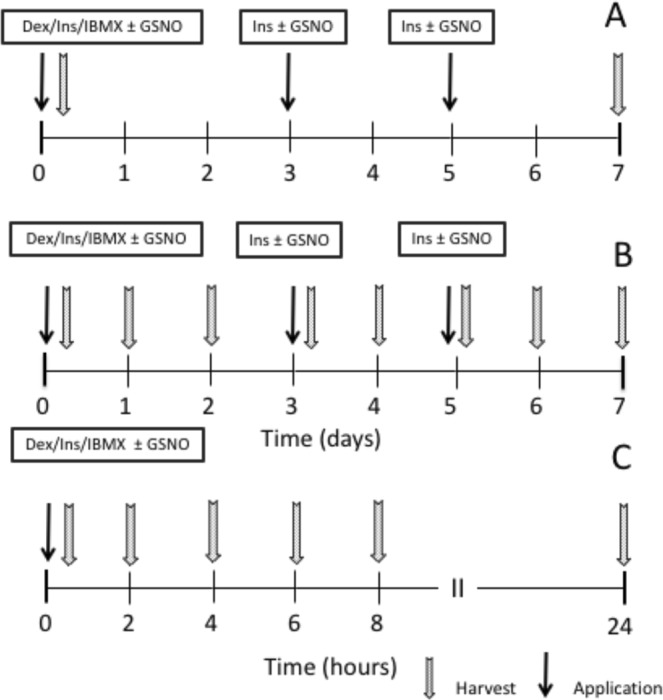
Differentiation protocols. Dex, dexamethasone; Ins, Insulin; IBMX; 3-Isobutyl-1-methylxanthine.

3T3-L1 preadipocytes (ATCC^®^ CL-173™) were cultured in Dulbecco’s Modified Eagle’s Medium (DMEM; high glucose) supplemented with 10% fetal bovine serum (FBS), penicillin and streptomycin at 37 °C in 5% CO_2_ atmosphere and 80% humidity. For standard experiments **(**protocol A; Fig. 10), cells were seeded onto 6-well plates and grown to confluence. Adipogenesis was induced 48 h *post* confluence (day 0) by addition of differentiation medium (culture medium containing 10 µg/ml insulin, 0.4 µg/ml dexamethasone, and 500 µM 3-isobutyl-1-methylxanthine; IBMX) in the absence and presence of GSNO or DETA/NO. At days 3 and 5, medium was replaced by fresh medium supplemented with 10 µg/ml and 0.2 µg/ml insulin, respectively, with and without NO donors. At day 7, adipogenesis was terminated by harvest of cells. In another approach, cells were differentiated for 7 days and harvested daily (protocol B). For short-term experiments (protocol C), cells were differentiated and harvested at 2, 4, 6, 8, and 24 h. For harvest, cells were treated with RIPA buffer (#R0278, Sigma) containing ethylenediaminetetraacetic acid (EDTA; 2 mM) and Complete Protease Inhibitor Cocktail (150 µl per well) and scraped off mechanically. Cell suspensions were transferred to Eppendorf vials, homogenized by repeated sonication, and kept on ice for 10 min. Aliquots were stored at 4 °C for measurement of TGs and total cellular protein content. For Western blot experiments homogenates were stored at −80 °.

### Cell viability

Viability of cells was measured as conversion of MTT to formazan using the Cayman MTT Cell Proliferation Assay Kit purchased through VWR International (Vienna, Austria). Briefly, cells were cultured and differentiated on 96-well plates as described. At day 7 of adipogenesis, medium was removed and cells were washed twice with phosphate-buffered saline (PBS). Thereafter, medium (100 µl) and MTT reagent (10 µl; prepared according to the manufacturer’s instruction) were added and cells incubated at 37 °C, 5% CO_2_, and 80% humidity for 3 h. Thereafter, medium containing excess MTT was removed carefully and formazan crystals were solubilised in 100 µl Crystal Dissolving Reagent supplemented with 10 µl HCl (10 mM). Formation of formazan was monitored at 550 nm using a Rosys Anthos ht3 photometer (Anthos Labtec Instruments GmbH, Salzburg, Austria). Cell-free medium was treated identically and used as blank^24^.

### Quantification of cellular protein and triglycerides

Protein concentration was determined using the Pierce^TM^ BCA Protein Assay Kit from ThermoFisher Scientific (Vienna, Austria) according to the manufacturer’s instructions. Briefly, cells were lysed in RIPA buffer (#R0278, Sigma) containing 2 mM EDTA, proteinase inhibitors (Complete®, Roche) and phosphatase inhibitors (PhosSTOP™, Roche) by repeated sonication on ice. Samples (25 µl) and standards (bovine serum albumin) were incubated with 200 µl reagent for 15 min at 37 °C and thereafter absorbance was measured at 562 nm using a SPECTROstar® Nano microplate reader (BMG LABTECH GmbH, Ortenberg, Germany). Cellular TGs were measured using the Infinity™ Triglycerides Liquid Reagent according to the manufacturer’s instruction using glycerol as standard. Results were expressed as nmol glycerol per mg protein.

### Trypan blue staining

Numbers of viable and non-viable cells were assessed by Trypan blue staining^25^. Briefly, 3T3-L1 cells were differentiated as described. At the indicated time points, cells were washed twice with PBS and trypsinized. Quantitative detachment of cells was examined microscopically. After centrifugation at 500 × g for 5 min, supernatants were aspired and cell pellets were resuspended in fresh medium. Aliquots of cell suspensions were diluted 1:1 in 0.4% Trypan blue solution. For counting of cells, this dilution (10 µl) was loaded on a hemocytometer. Viable and non-viable cells were counted in every corner square of the Neubauer chamber and total cell number as well as the percentage of non-viable cells were calculated as described^25^.

### Analysis of mRNA expression

Total RNA was isolated using the GenElute^TM^ Mammalian Total RNA Miniprep Kit (ThermoFisher Scientific) including DNAse I treatment of samples to eliminate genomic DNA. Quality of RNA was determined by measuring OD_260_/OD_280_ by UV/Vis spectroscopy using a Nanodrop 2000 spectrophotometer (VWR International GmbH). RNA was transcribed to cDNA using the High Capacity cDNA Reverse Transcription Kit (ThermoFisher Scientific). Real-time PCR analysis was performed with ~10–30 ng of cDNA using TaqMan^®^ Universal PCR Master Mix and pre-designed TaqMan^®^ Gene Expression Assays (Supplemental Table 2). Reactions were carried out on a StepOnePlus^TM^ Real-Time PCR System (ThermoFisher Scientific). Cycling conditions were as follows: 2 min at 50 °C, 10 min at 95 °C, 40 cycles of 15 s at 95 °C and for 1 min at 60 °C. Data were analyzed according to the 2^−∆∆Ct^ method using cyclophilin D as reference gene. Lack of amplification was verified in no-template controls.

### Western blot analysis

Homogenates were denaturated by boiling with 5-fold Laemmli buffer for 10 min at 95 °C. Samples containing 10–20 µg of protein were separated by sodium dodecyl sulfate polyacrylamide gel electrophoresis on 10% or 12% gels for 45 min at 180 V. Thereafter, proteins were transferred onto nitrocellulose membranes for 90 min (240 mA). After blocking with Tris-buffered saline containing 0.1% (v/v) Tween-20 and 5% non-fat dry milk for 1 h (ambient temperature) membranes were incubated overnight at 4 °C with primary antibodies (Supplemental Table 1). After incubation of membranes with respective horseradish peroxidase-conjugated anti-rabbit or anti-mouse IgGs (1:5,000) immunoreactive bands were visualized using Western Bright^TM^ ECL or Western Bright^TM^ Quantum (Biozym, Vienna, Austria) and chemiluminescence was quantified with the Fusion SL Imaging System (VWR International GmbH).

### Preparation of nuclear-enriched fractions

Adipogenesis of cells (grown on 100 mm dishes) was induced in the presence or absence of GSNO (1 mM). For biotin switch experiments, cells were scraped off mechanically 6 h after induction using 1 ml of HEN-buffer (pH 7.8) composed of 100 mM 4-(2-hydroxyethyl)-1-piperazineethanesulfonic acid (HEPES), 1 mM EDTA, 0.1 mM neocuproine, Complete Protease Inhibitor Cocktail, and Phosphatase Inhibitor Cocktail. After centrifugation at ambient temperature for 5 min at 1,000 × g, cell pellets were incubated in HEN-buffer supplemented with 0.2% Igepal CA-630 for 3 min on ice. After centrifugation for 5 min at 1,000 × g and 4 °C, supernatants (cytosolic fractions) were collected. Pellets (nuclear fractions) were resuspended in 150 µl HEN buffer and homogenized by sonication for 3 s on ice. Cytosolic and nuclear fractions were stored at −80 °C.

### Detection of S-nitrosated C/EBPβ and C/EBPδ by biotin switch

S-nitrosation of C/EBPβ and C/EBPδ was visualized by the biotin switch method^26^. Nuclear pellets (100 µg) were incubated with 42 mM S-methyl methanethiosulfonate and 2.5% SDS in HEN-buffer (pH 7.8) for 30 min at 55 °C to saturate protein sulfhydryl groups. Nuclear proteins were precipitated with iced acetone at −20 °C for 60 min and thereafter centrifuged for 10 min at 4 °C and 2,000 × g. Pellets were washed with 80% acetone and then resuspended by sonication in HEN-buffer supplemented with 1% SDS. For biotinylation, samples were treated with 0.46 mM pyridyldithiol-activated biotin (#21341; ThermoFisher Scientific) in the absence and presence of 30 mM sodium ascorbate for 90 min at ambient temperature in the dark. Proteins were precipitated and pellets were washed as described above. Thereafter, pellets were resuspended in 250 µl of 10 mM HEPES buffer containing 1% SDS. The solution was neutralized with 750 µl of 25 mM HEPES buffer containing 100 mM NaCl, 1 mM EDTA, and 1% Triton X-100 (pH 7.5). Samples were incubated overnight at 4 °C with 40 µl of streptavidin-agarose beads (#E5529, Sigma). Thereafter, beads were treated 4 times with 1 ml of 25 mM HEPES buffer containing 600 mM NaCl, 1 mM EDTA, and 1% Triton X-100 and then centrifuged for 90 s at ambient temperature and 8,000 × g. Pellets were washed twice and then centrifuged as described. Beads were boiled in 2-fold Laemmli buffer for 10 min at 95 °C and denatured proteins were subjected to Western blot and probed for C/EBPβ and C/EBPδ proteins.

### Transcriptional activity of C/EBPβ

Transcriptional activity of C/EBPβ was measured using the C/EBP Cignal^TM^ Reporter Assay (CCS-001L; dual luc) from Qiagen (Hilden, Germany) and the Dual-Luciferase® Reporter Assay System (E1910; Promega Corporation, Mannheim, Germany). pcDNA-mC/EBPβ (provided as bacterial agar stab) was a gift from Jed Friedman (Addgene plasmid # 49198). Bacteria were precultured overnight in lysogeny broth medium containing ampicillin (100 µg/ml). Thereafter, plasmid DNA of a 100 ml culture was isolated using PureLink™ HiPure Plasmid Filter Maxiprep Kit (ThermoFisher Scientific) and sequenced for C/EBPβ insert by Microsynth AG (Balgach, Switzerland). HEK 293 cells were cultured in poly-L-lysine-coated 6-well plates using DMEM (high glucose) supplemented with 10% FBS, 2 mM glutamine, 1% penicillin, and 1% streptomycin at 37 °C, 5% CO_2_, and 80% humidity. At 80–90% confluence, cells were co-transfected with C/EBPβ DNA (1 µg) and Cignal Reporter (0.5 µg) in serum- and antibiotic-free medium using Metafectene® Pro (Biontex Laboratories GmbH; München, Germany). Positive and negative control transfections were performed with constructs provided with the kit. Cells were incubated for 48 h with medium changes every 24 h. Transfections were performed in the absence and presence of 100 µM DETA/NO. Thereafter, cells were harvested and lysed according to the manufacturer´s instructions. Activities of (inducible) firefly luciferase and (constitutively expressed) *Renilla* luciferase were sequentially measured as bioluminescence using the SpectraMax® GEMINI EM Microplate Spectrofluorometer (Molecular Devices, Wals-Siezenheim, Austria). Luminescence of each sample was normalized to *Renilla* luciferase activity.

### Oil Red O Staining of lipids

Accumulation of TGs was visualized by Oil Red O staining^27^. Cells were cultivated on microscope cover glasses (Ø: 3 cm), placed in cell culture dishes, and differentiated according to protocol A. At day 7, cells were washed with PBS and thereafter treated with 2 ml of formalin (10%) in PBS. After fixation for 10 min, coverslips were stored in fresh formalin solution protected from light and evaporation until further use. Before staining, cells were washed twice with Milli-Q water and incubated with isopropanol (60%) for 5 min. Then, coverslips were dried completely. Staining was performed with 1 ml of Oil Red O working solution (0.2% in 60% isopropanol) for 10 min followed by intense washing with Milli-Q water. Cells were visualized using an Olympus CKX41 microscope. Thereafter, the dye was eluted with isopropanol (100%), incubated for 10 min under gentle shaking, and quantified spectroscopically at 500 nm.

### Nile red staining of lipids

For Nile Red staining, formalin-fixed cells were washed twice with distilled water, followed by incubation with 60% isopropanol for 5 min at ambient temperature. Thereafter, isopropanol was removed and cells were dried. Then, cells were incubated with Nile Red (1 µM in PBS) for 10 min at ambient temperature and protected from light. After extensive washing, cells were treated with 4′,6-diamidino-2-phenylindole dihydrochloride (DAPI; 100 ng/ml in PBS) as a nuclear counterstain (5 min; ambient temperature; light-protected). After extensive washing Nile Red and DAPI fluorescence were visualized by an Axiovert 200 M microscope (Carl Zeiss GmbH, Vienna, Austria) using a Green Fluorescent Protein filter (λ_Ex_: 450–500 nm; λ_Em_: 528 nm) and a DAPI-filter (λ_Ex_: 395 nm; λ_Em_: 460 nm), respectively.

### Quantification of cellular S-nitrosothiols

Endogenous S-nitrosothiols were determined as HgCl_2_-sensitive formation of nitrite^28^ using the fluorophore 2,3-diaminonaphthalene (DAN)^29^. Differentiation of 3T3-L1 cells (grown on 100 mm plates) was induced in the absence and presence of GSNO (1 mM) as described. At the indicated time points (0, 1 h, 3 h, 6 h) cells were washed 4-fold with PBS, scraped off mechanically in ice-cold PBS, and homogenized by sonication on ice. Homogenates were centrifuged at 20,000 × g for 10 min at 4 °C and supernatants were used for further experiments. For quantification of S-nitrosothiols, samples were incubated with DAN (final concentration 26 µM in 0.62 M HCl) in the absence or presence of HgCl_2_ (200 µM) for 45 min at 37 °C. Reaction mixtures were protected from light. To maximize fluorescence, samples were adjusted to pH 11.5–12.0 by addition of NaOH (10 µl; 2.8 M) and then kept at ambient temperature for further 10 min. Fluorescence was measured using a Microplate Spectrofluorometer (Spectra Max Gemini EM; Molecular Devices, Germany) with excitation and emission wavelengths of 355 nm and 405 nm, respectively. S-Nitrosothiol concentrations were calculated as HgCl_2_-sensitive formation of nitrite by comparison to a standard curve.

### Data analysis and statistics

Statistical analysis was performed using the KaleidaGraph® software version 4.1.3 from Synergy software (Reading, PA, USA). Statistical significance between two groups was analyzed using the unpaired t-test with equal variance. To judge for statistical significance between more than two groups, analysis of variance (ANOVA) was performed using Student-Newman-Keuls as *post-hoc* test. For analysis of qPCR experiments, statistical significance was tested using ΔCt values. A probability (p) value < 0.05 was considered statistically significant. Data were expressed as mean values ± standard error of the mean (SEM).

## Supplementary information

Supplemental Information & Original Western Blots

## Data Availability

All materials, data and associated protocols will be available to readers without undue qualifications in material transfer agreements.
