# Author Correction: S-nitrosoglutathione inhibits adipogenesis in 3T3-L1 preadipocytes by S-nitrosation of CCAAT/enhancer-binding protein β

**DOI:** 10.1038/s41598-020-67063-w

**Published:** 2020-06-12

**Authors:** Marion Mussbacher, Heike Stessel, Teresa Pirker, Antonius C. F. Gorren, Bernd Mayer, Astrid Schrammel

**Affiliations:** 1 Department of Pharmacology and Toxicology, University of Graz, Humboldtstraße 46, A-8010 Graz, Austria; 2 Center for Physiology and Pharmacology, Department of Vascular Biology and Thrombosis Research, Medical University of Vienna, Schwarzspanierstraβe 17, A-1090 Vienna, Austria

Correction to: *Scientific Reports* 10.1038/s41598-019-51579-x, published online 28 October 2019

This Article contains errors.

As a result of an error during figure assembly, in Figure 3F the images for Nile Red/DAPI control and DETA/NO are duplicates of Figure 2A Nile Red/DAPI images for control and GSNO, respectively. The corrected Figure 3F is included below as Figure 1.

**Figure 1:**
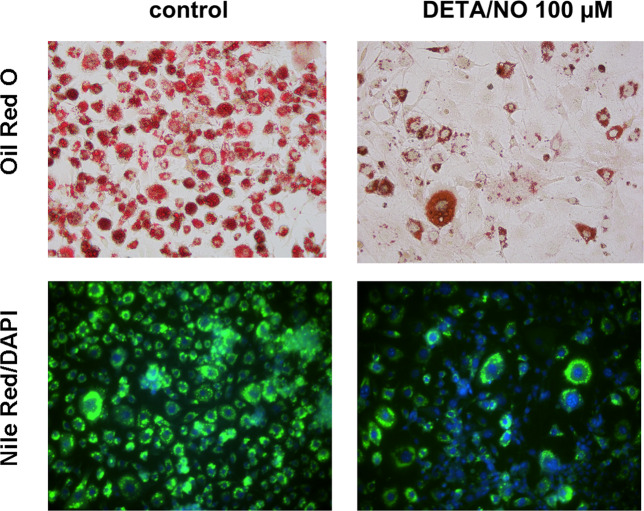
.

These corrections do not affect the conclusions of the Article.

Additionally, the raw data for all replicates for Figures 2A and 3F are now shown in the Supplementary Information file included below.

## Supplementary information

Supplementary Information.

