# Supplementary material for: S-nitrosoglutathione inhibits adipogenesis in 3T3-L1 preadipocytes by S-nitrosation of CCAAT/enhancer-binding protein β
Source: Sci Rep. 2019 Oct 28;9:15403. doi: 10.1038/s41598-019-51579-x (PMC6817858; doi:10.1038/s41598-019-51579-x)
Supplement: Supplementary file 1 — Supplemental Information & Original Western Blots [file 41598_2019_51579_MOESM1_ESM.pdf]

SUPPLEMENTAL MATERIAL

S-nitrosoglutathione inhibits adipogenesis in 3T3-L1 preadipocytes by S-nitrosation of CCAAT/enhancer-binding protein β

Marion Mussbacher[1,2], Heike Stessel[1], Teresa Pirker[1], Antonius C.F. Gorren[1], Bernd Mayer[1], and Astrid Schrammel[1*]

[1] Department of Pharmacology and Toxicology, University of Graz, Humboldtstrasse 46, A-8010 Graz, Austria

[2] Center for Physiology and Pharmacology, Department of Vascular Biology and Thrombosis Research, Medical University of Vienna, Schwarzspanierstraβe 17, A-1090 Vienna, Austria

* Corresponding author

Antibody	Dilution	Company	Catalogue #
AMPKα	1:1,000	Cell Signaling	2532
ATGL	1:1,000	Cell Signaling	2138
β-actin	1:300,000	Sigma	1978
Caspase-3	1:1,000	Cell Signaling	9665
C/EBPβ	1:1,000	ThermoFisher Scientific	MA1-827
C/EBPδ	1:1,000	Cell Signaling	2318
CGI-58	1:2,000	Novus Biologicals	NB 110-41576
GAPDH	1:40,000	Sigma	G 8795
HSL	1:1,000	Cell Signaling	4107
$pThr^{172}$-AMPKα	1:2,000	Cell Signaling	2535
$pThr^{188}$-C/EBPβ	1:2,000	Cell Signaling	3084
PARP	1:1,000	Cell Signaling	9542
PPARγ (1,2)	1:1,000	Cell Signaling	2430
SP-1	1:1,000	Millipore	ABE135
SREBP-1	1:1,000	Abcam	ab3259

Table 1: List of antibodies in alphabetical order.

**Gene symbol**	**TaqMan® Assay**
C/EBPα	Mm00514283_s1
C/EBPβ	Mm00843434_s1
C/EBPδ	Mm00786711_s1
Cyclophilin D	Mm00835365_g1
FABP4	Mm00445878_m1
IL-6	Mm00446190_m1
Leptin	Mm00434759_m1
LPL	Mm01345523_m1
PPARγ (2)	Mm01184322_m1
SREBP-1 (a,c)	Mm00550338_m1

Table 2: Pre-designed TaqMan® Gene Expression Assays in alphabetical order.

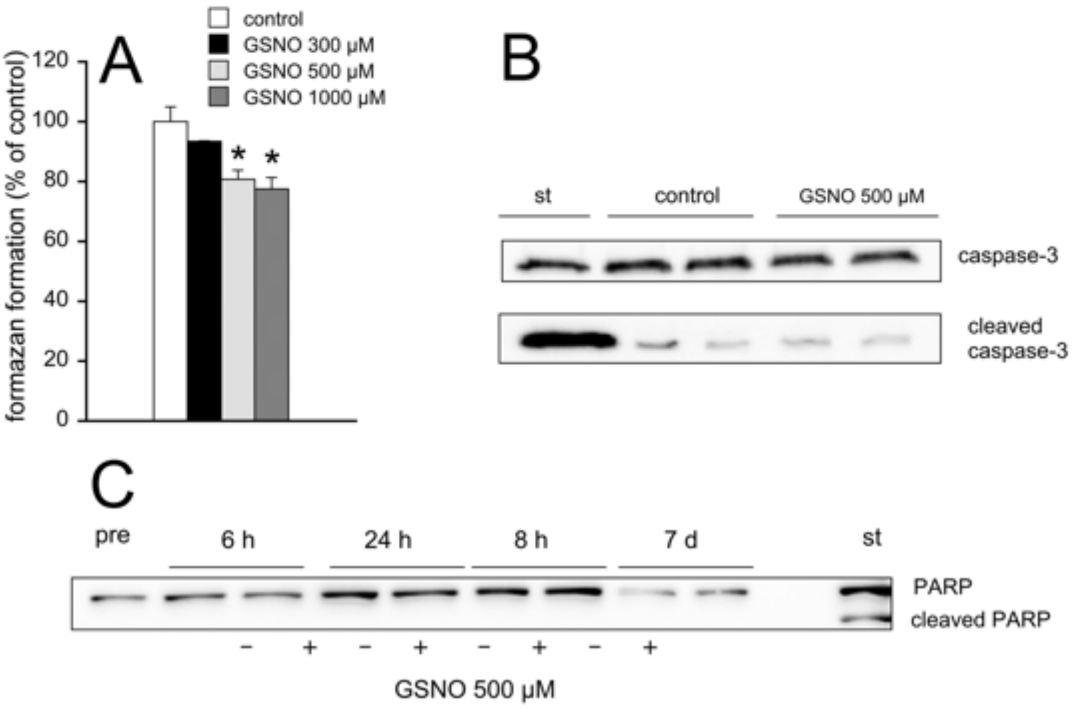

Supplemental Figure S1 Cytotoxicity of GSNO. 3T3-L1 cells were differentiated in the absence and presence of GSNO (500 μM) according to protocol A. Cell viability was measured using the MTT test (A). Formazan formation was slightly lowered at GSNO concentrations ≥500 μM. Data represent mean values ± SEM of 3 individual experiments. Protein expression of caspase-3 and cleaved caspase 3 (B) as well as PARP and cleaved PARP (C) was measured in whole-cell lysates by Western blot. Cells that have been treated with staurosporin for 5 h served as positive control; pre, preadipocytes; st, staurosporine.

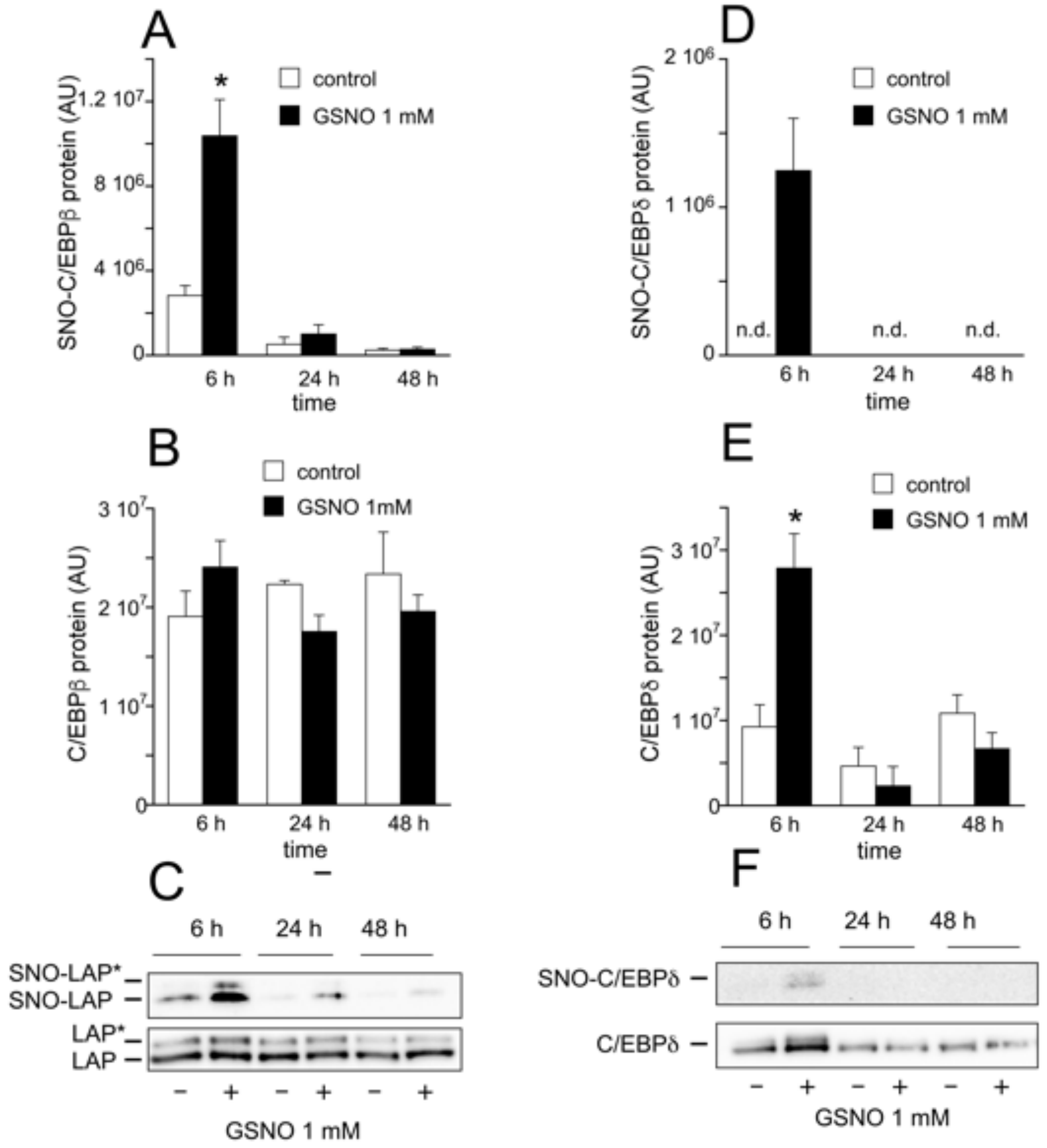

Supplemental Figure S2 Time course of C/EBPβ and C/EBPδ S-nitrosation. 3T3-L1 cells were differentiated in the absence and presence of GSNO (1 mM) up to 48 h. S-nitrosation was analyzed in crude nuclear extracts by biotin switch. S-nitrosation of C/EBPβ isoforms LAP* and LAP was maximal at 6 h but was also detectable 24 h and 48 h (A). Nuclear expression of total C/EBPβ protein was not significantly affected over 48 h (B). Data represent mean values ± SEM of 3-8 individual experiments. Representative Western blots (C). S-nitrosation of C/EBPδ was hardly detectable at 6 h of differentiation and absent at later periods of differentiation (D) Of note, the signal was obtained with maximal amplification and exposure time. Interestingly, after 6 h of differentiation, protein expression of C/EBPδ was significantly upregulated in the presence of GSNO (1 mM; E). Data represent mean values ± SEM of 3 individual experiments; *p<0.05 *vs* untreated control; Representative Western blots (F).

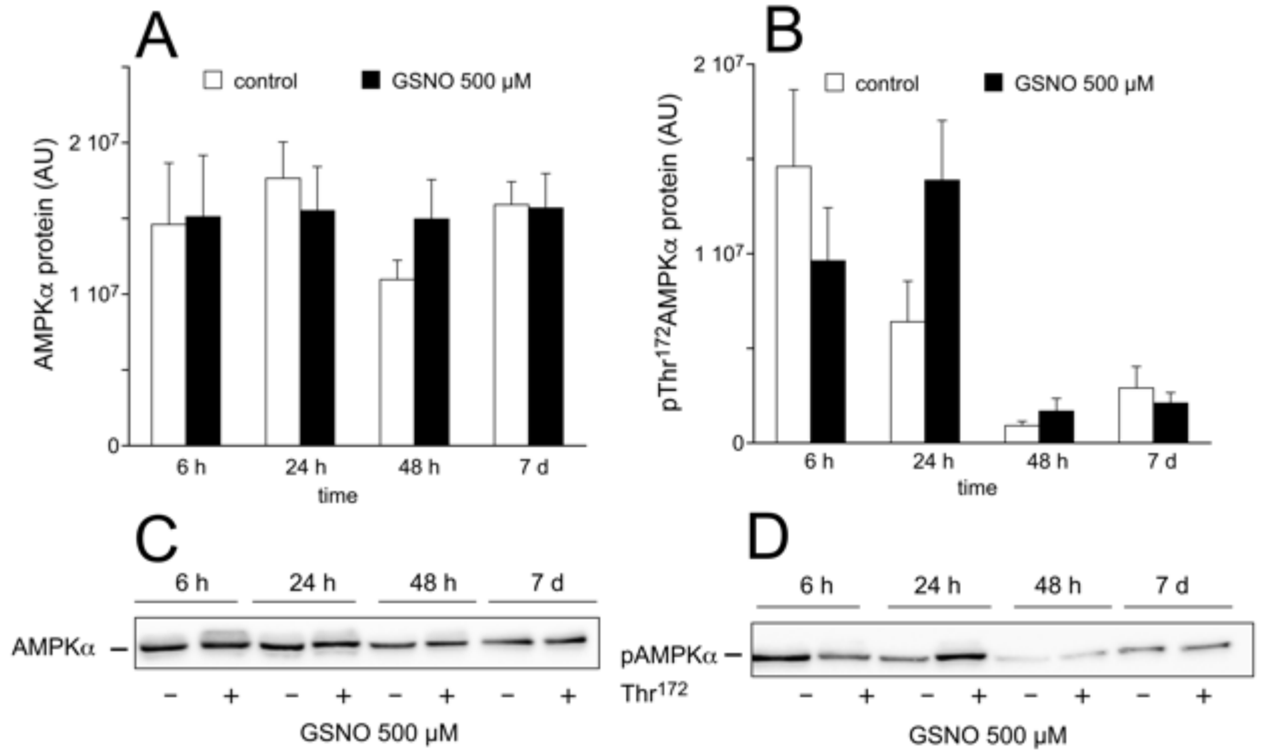

Supplemental Figure S3 Effect of GSNO on AMPK protein expression and phosphorylation. 3T3-L1 cells were differentiated up to 7 days in the absence and presence of GSNO (500 μM). Protein expression (A, C) and phosphorylation of AMPK at threonine 172 (B, D) were analyzed at indicated time points in whole-cell lysates. GSNO exerted no significant effect. Data represent mean values ± SEM of 4 individual experiments.

Original Westernblots

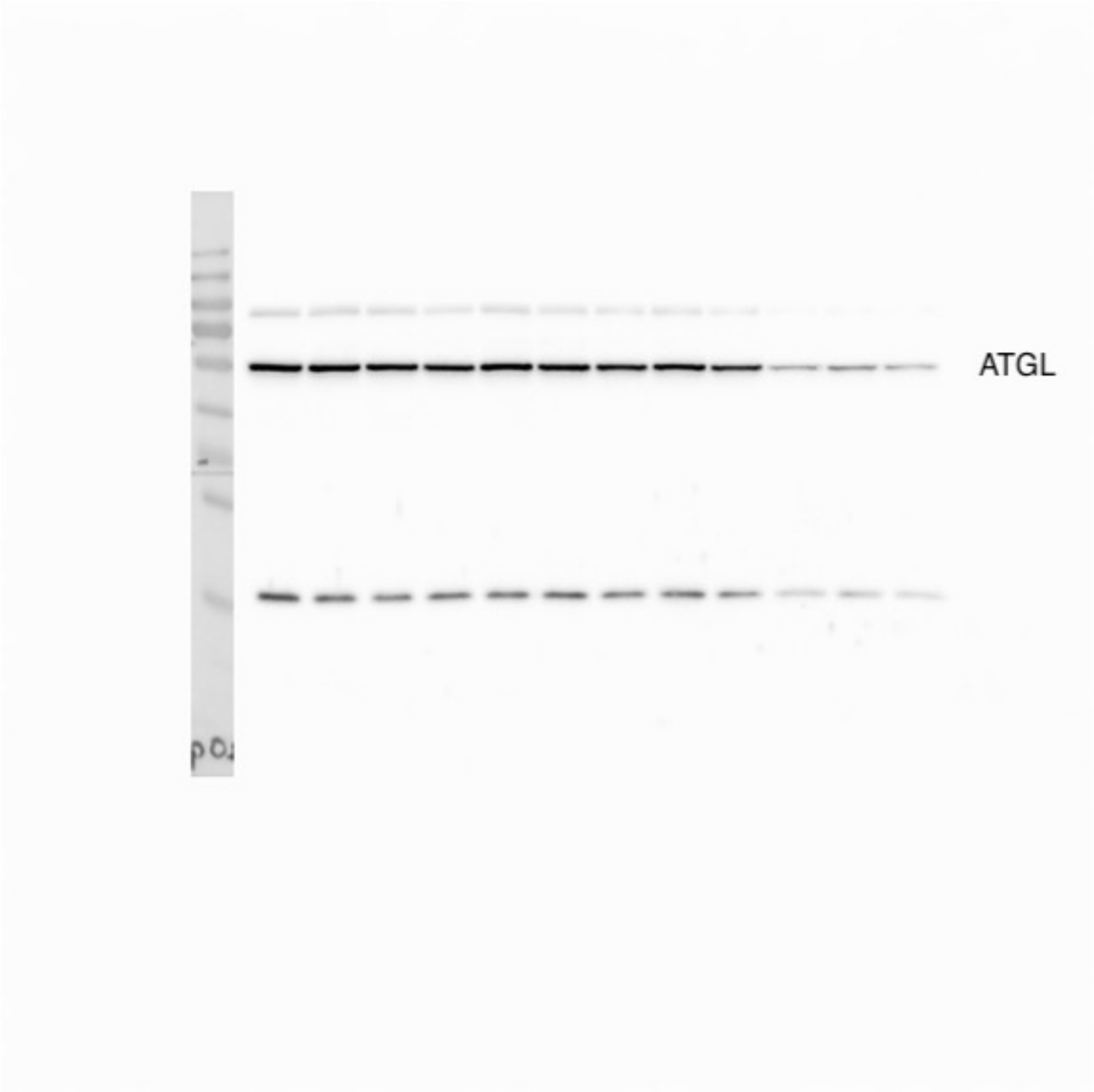

Fig. 1F_ATGL

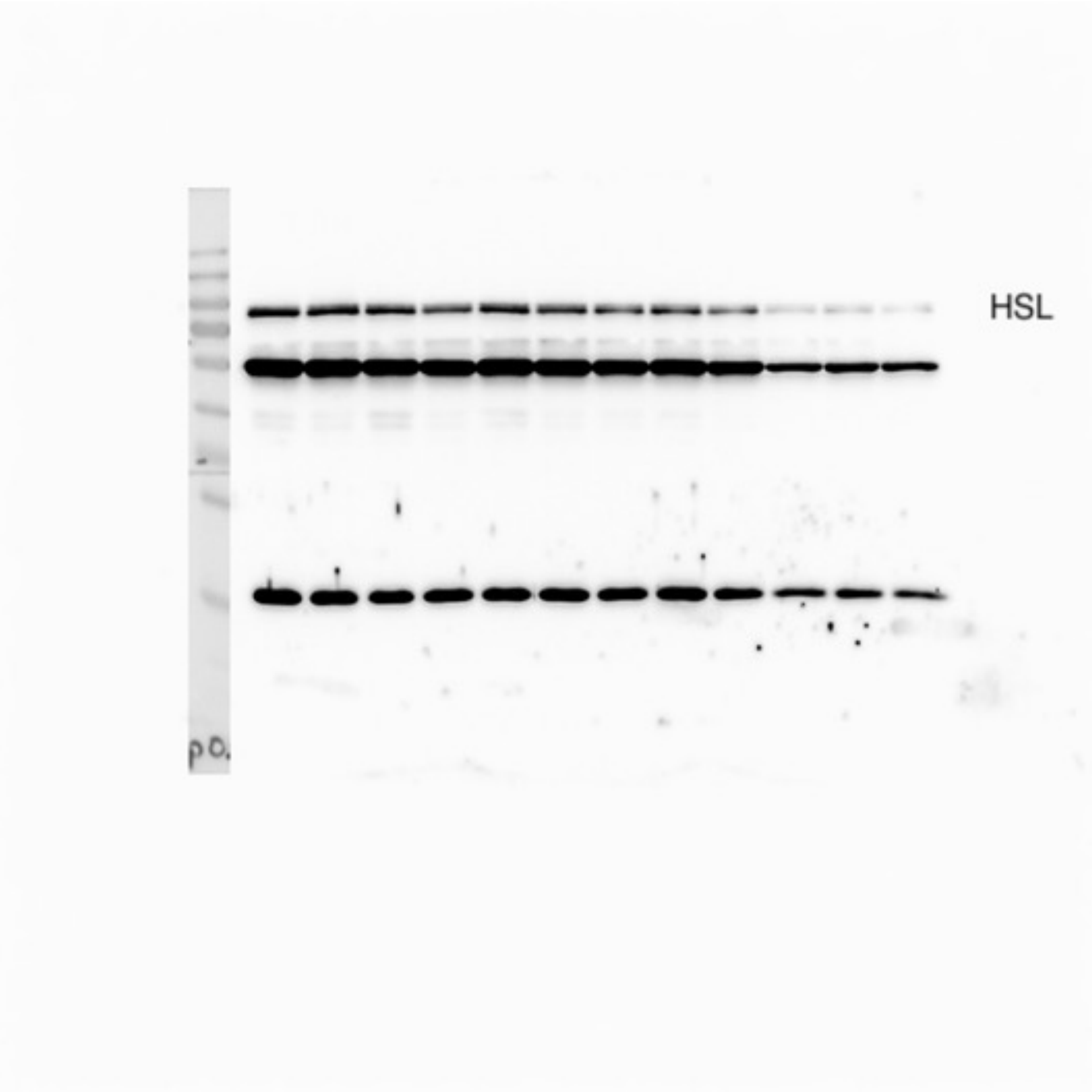

Fig. 1F_HSL

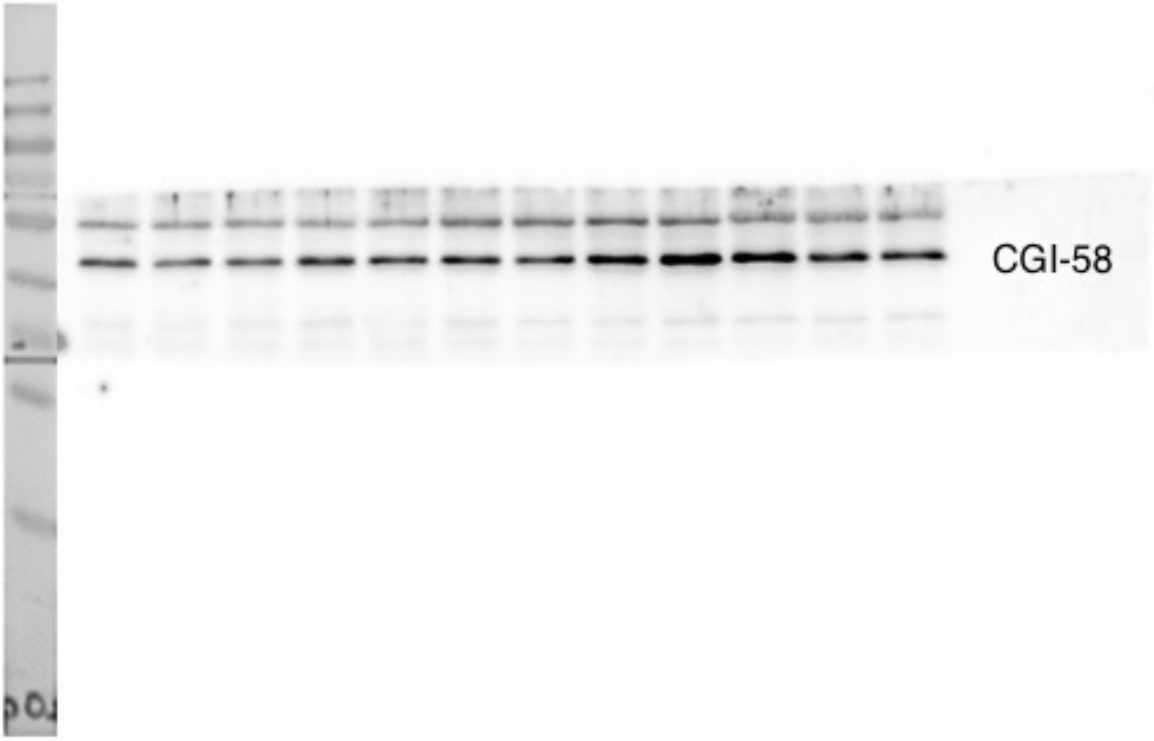

Fig. 1F_CGI-58

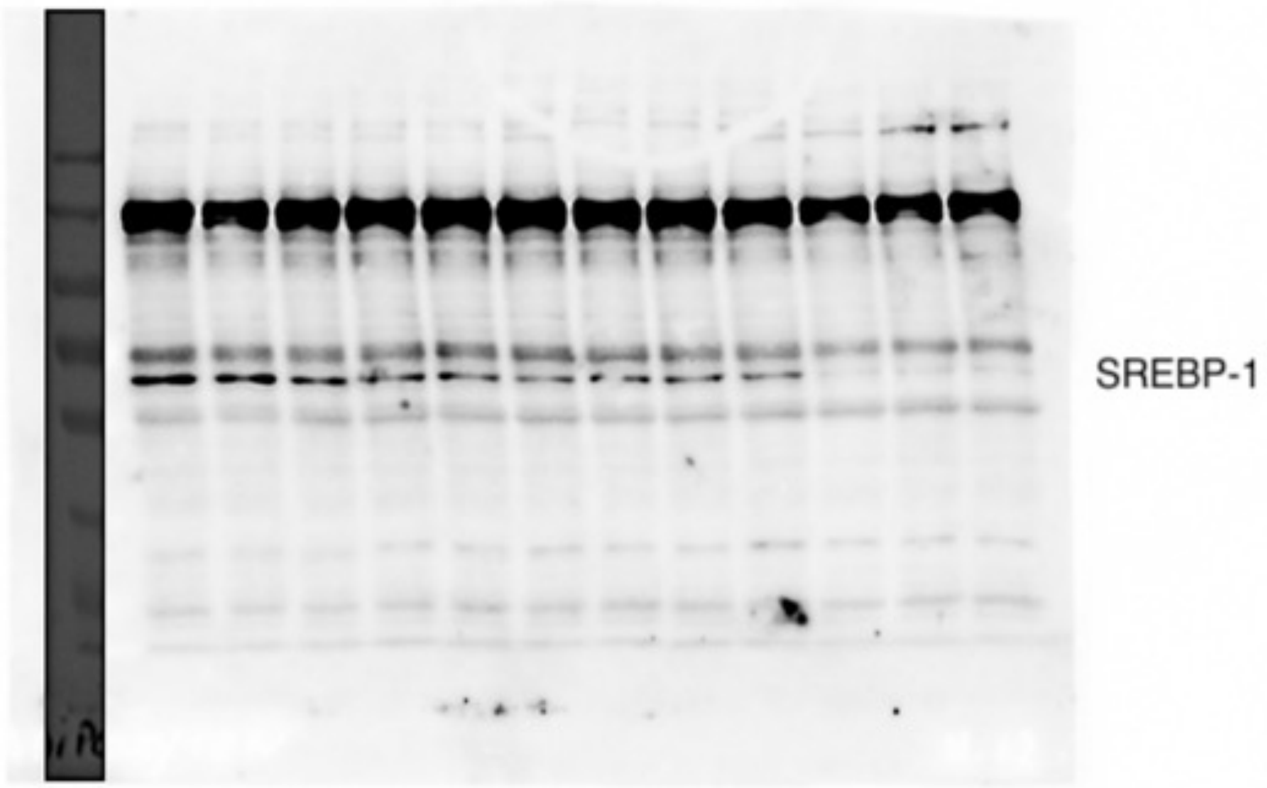

Fig. 1F_SREBP-1

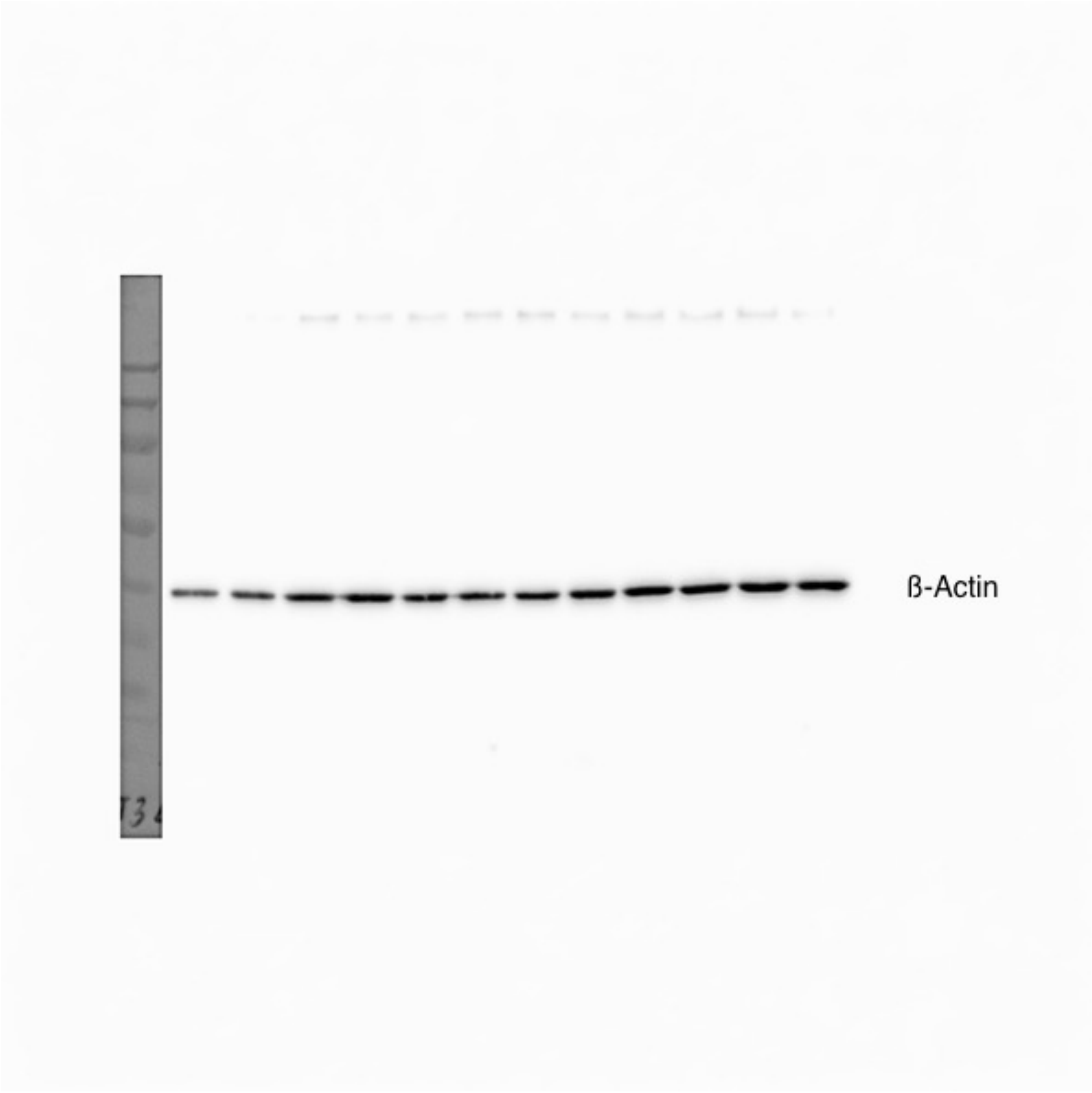

Fig. 1F_ß-actin

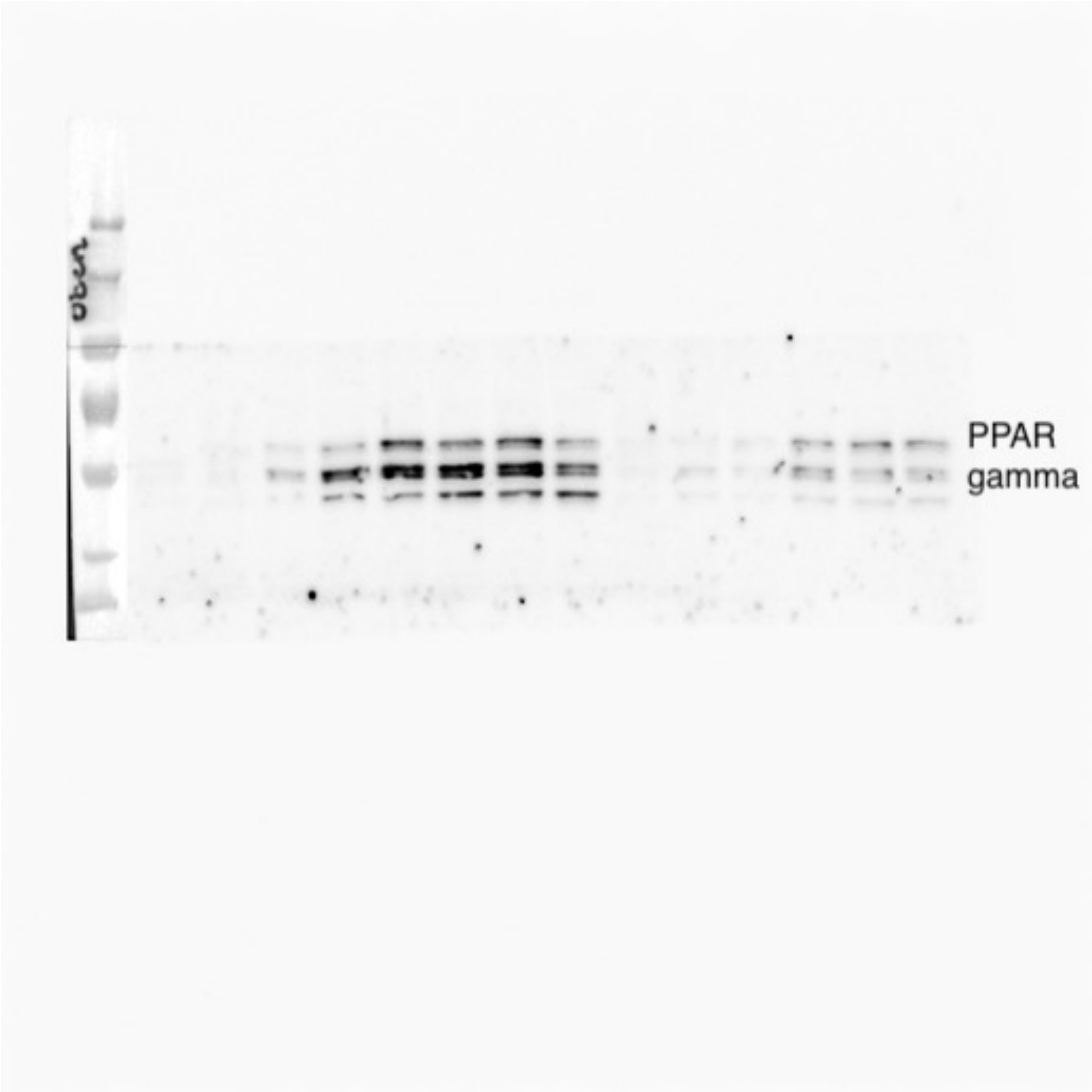

Fig 5H_PPARγ

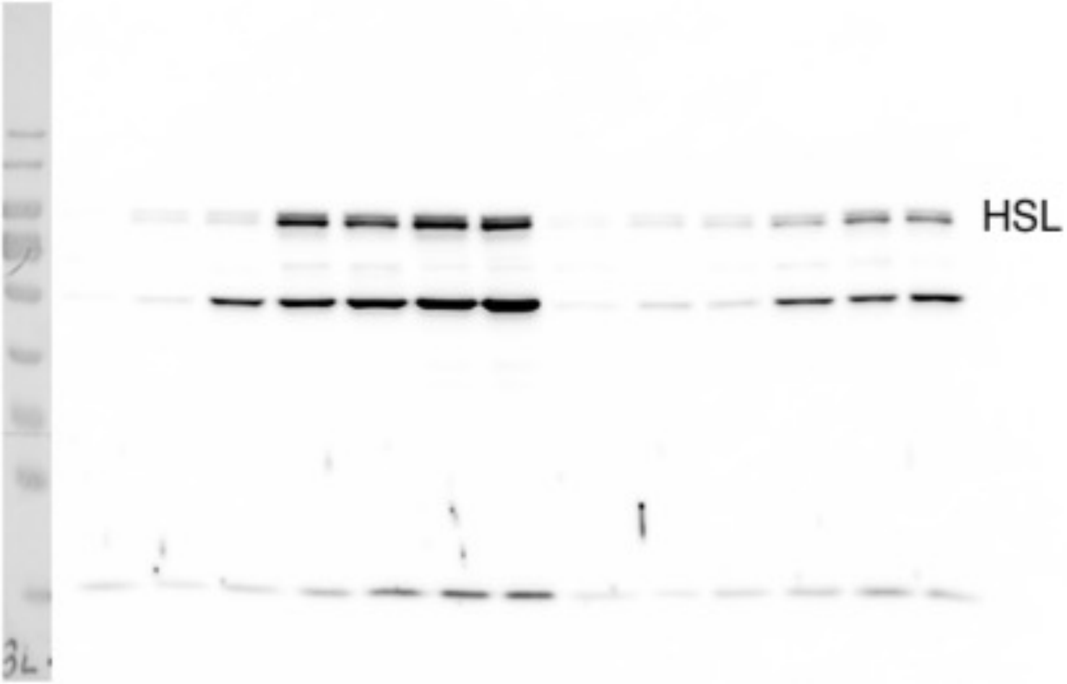

Fig 5H_HSL

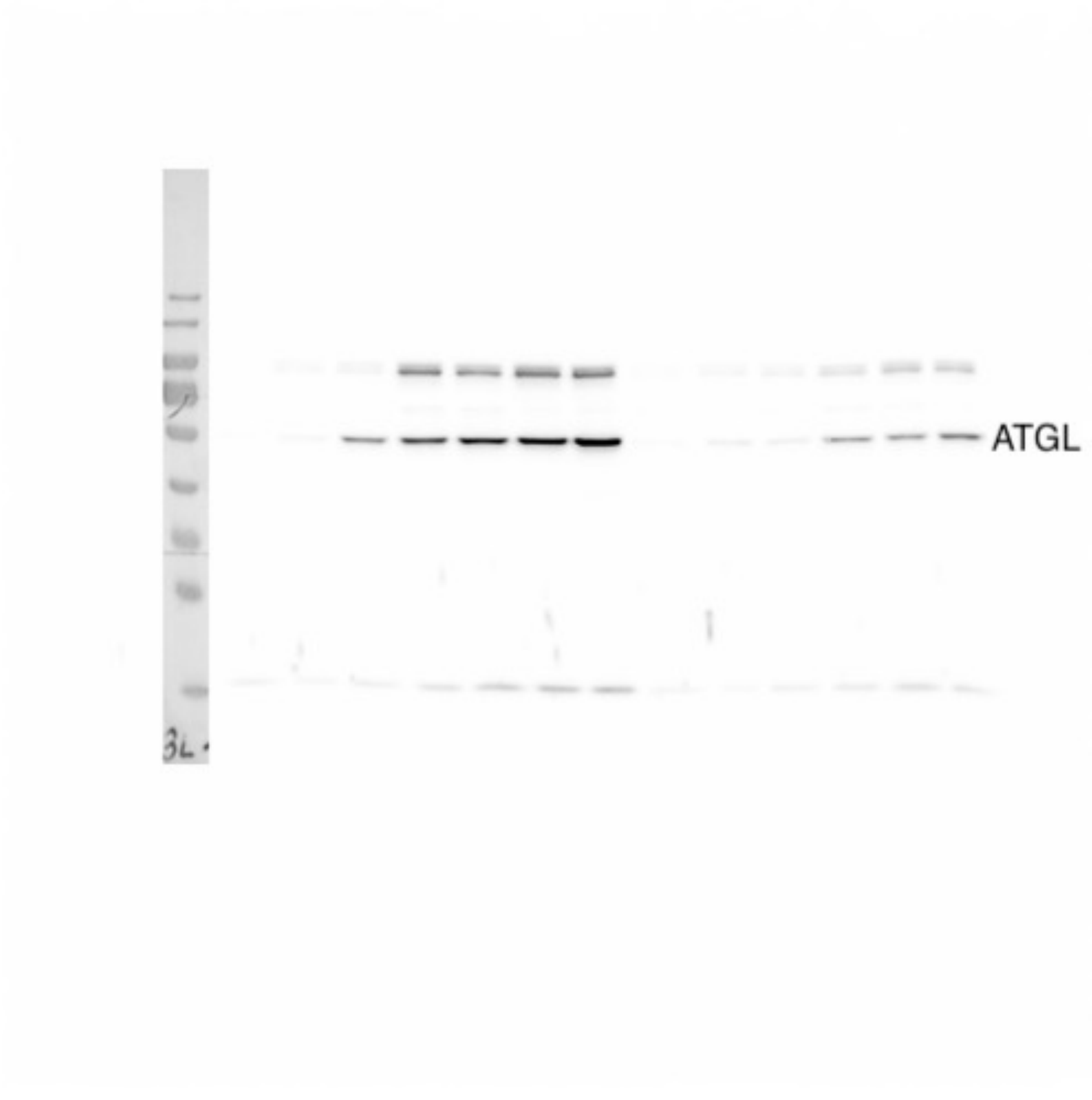

Fig 5H_ATGL

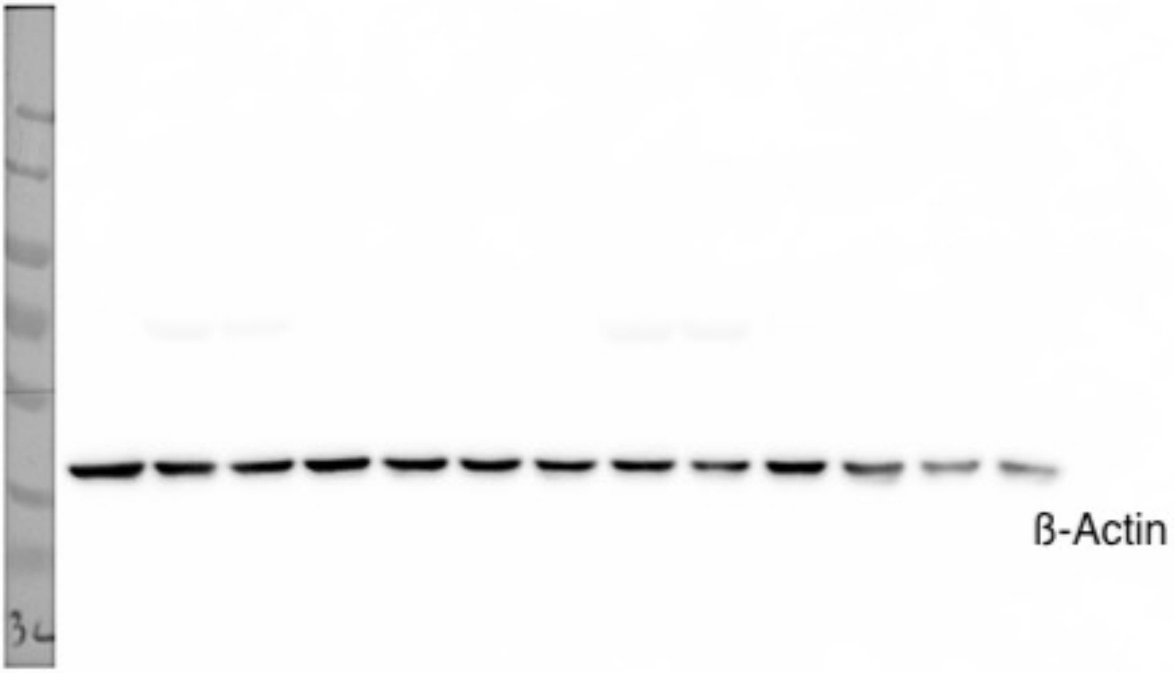

Fig 5H_β -actin

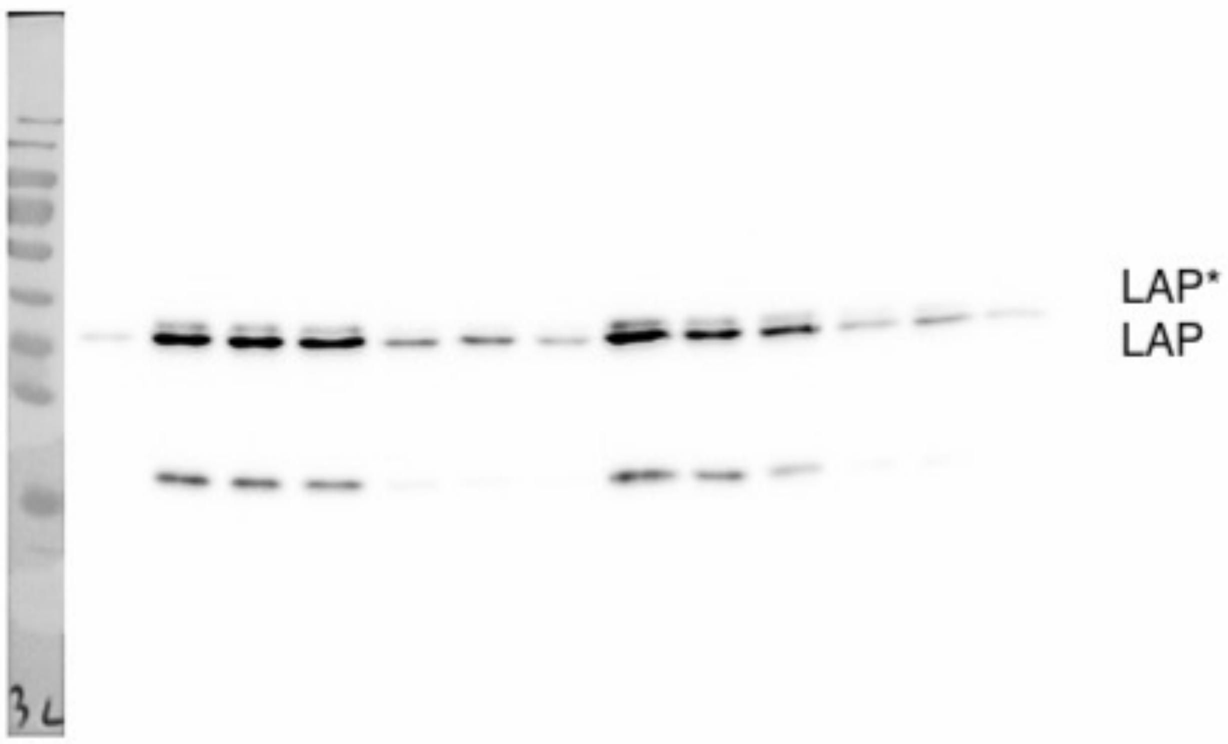

Fig 5H_CEBP/β_LAP* & LAP

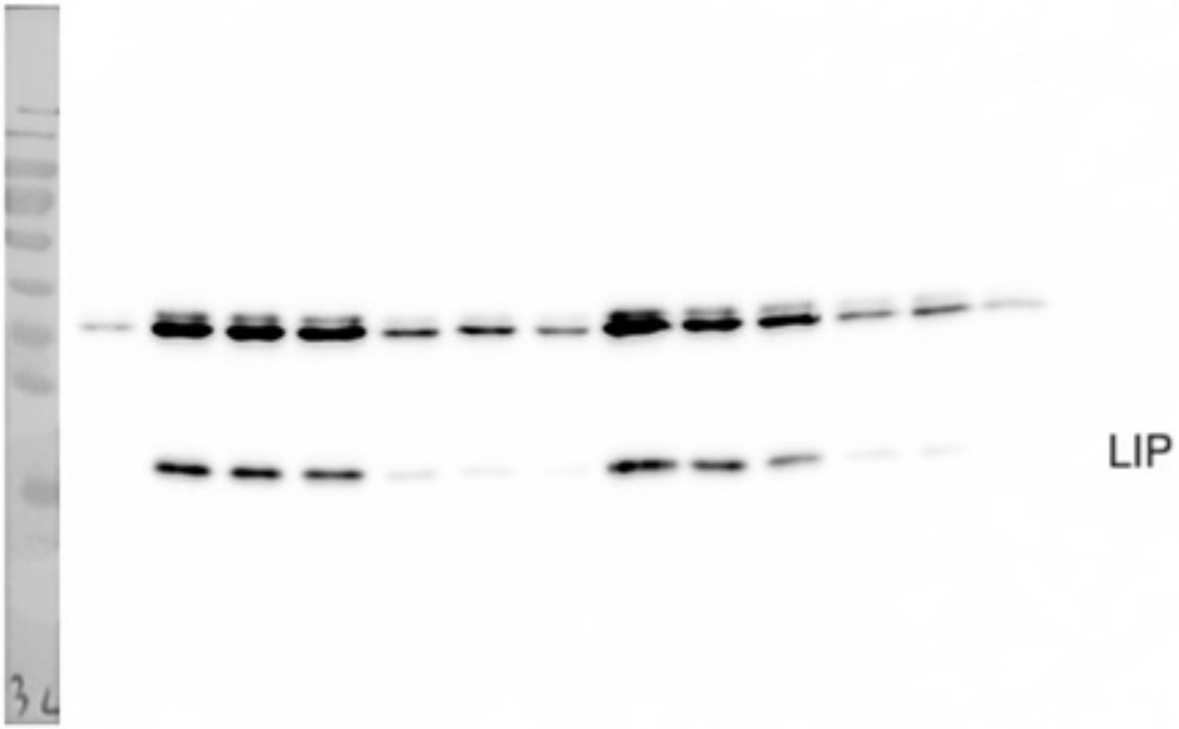

Fig 5H_CEBP/β_LIP

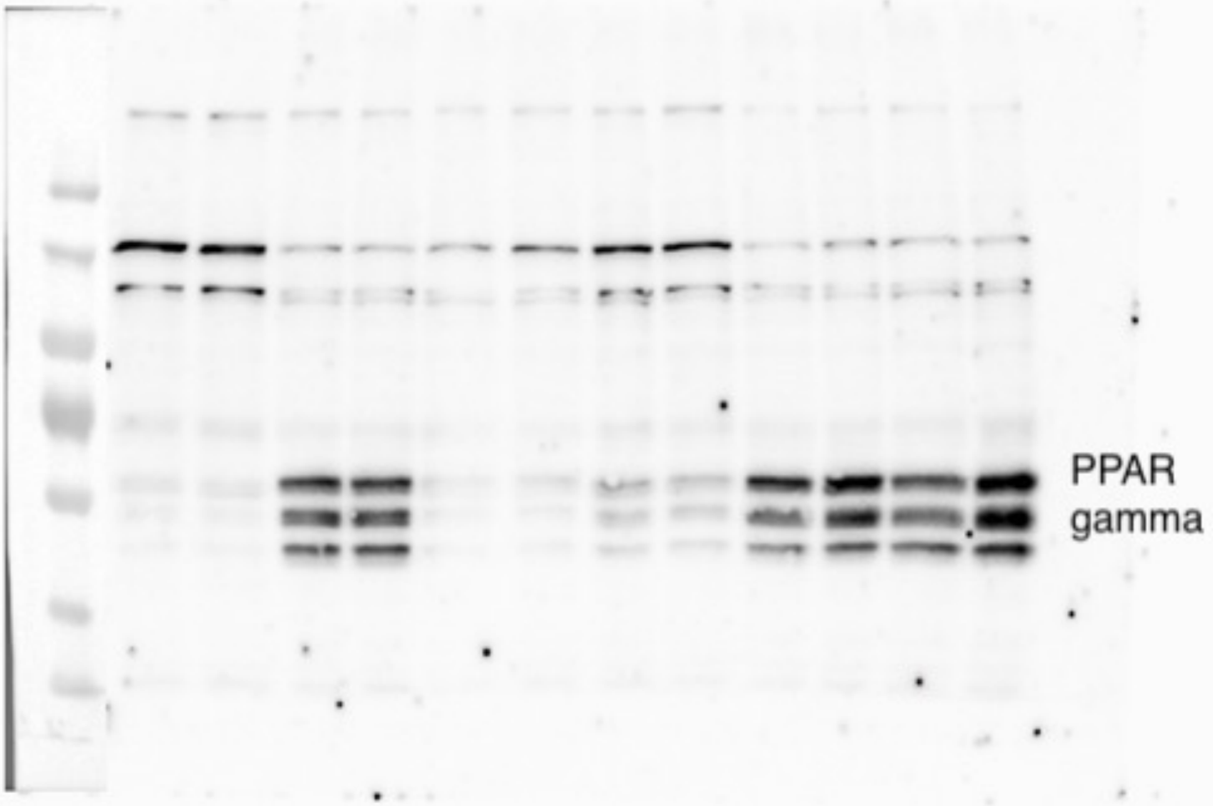

Fig. 7B_ PPARϒ

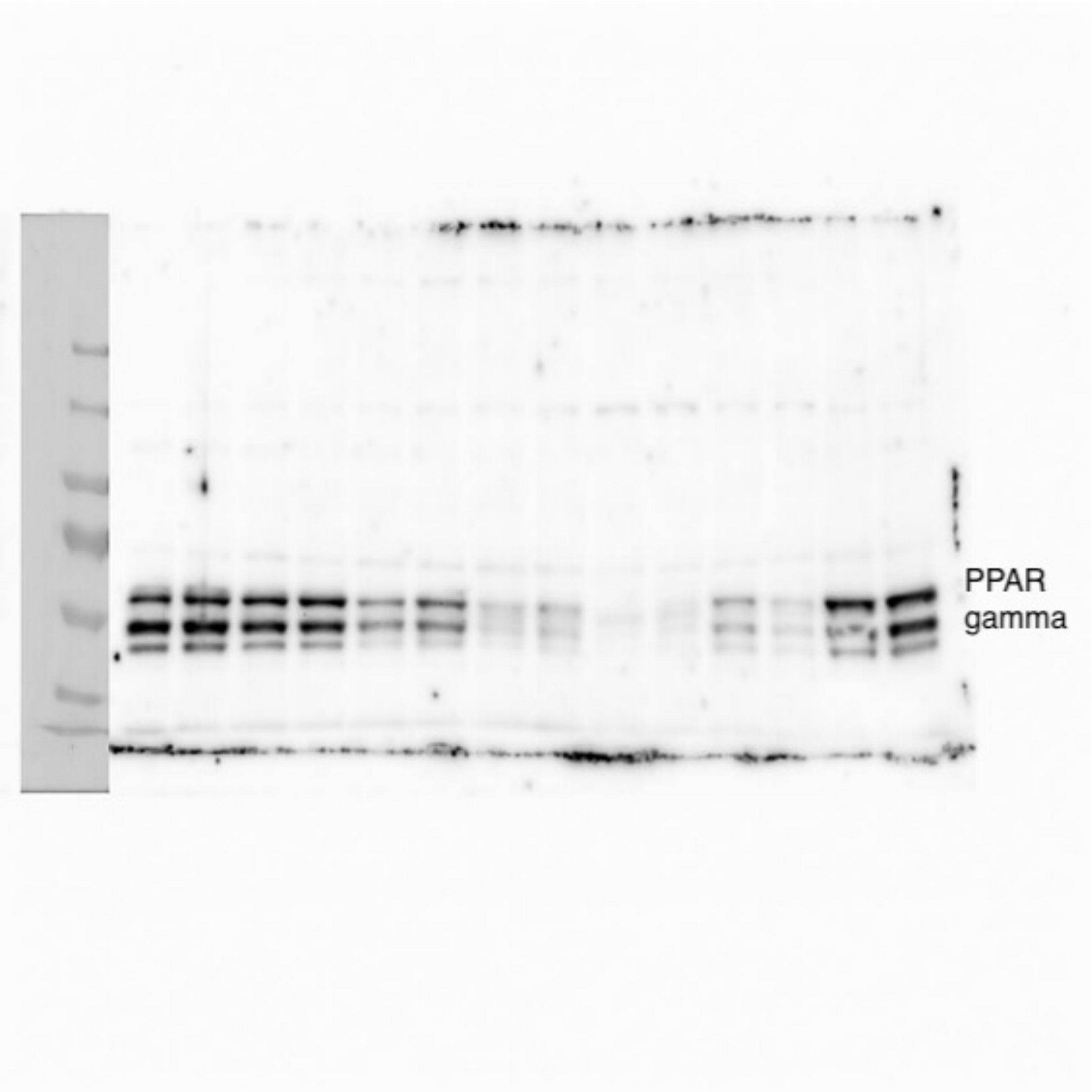

Fig. 7D_ PPARϒ

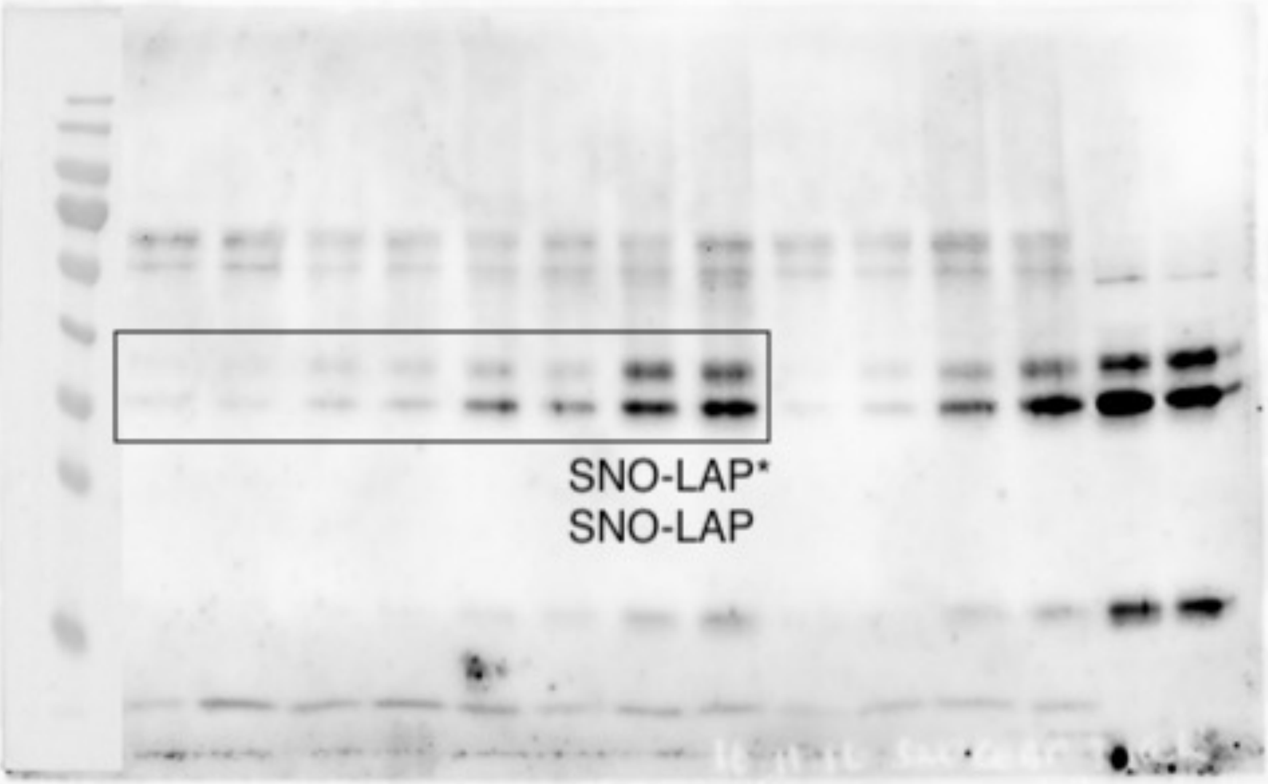

Fig. 8A_SNO-C/EBPβ_LAP* &LAP

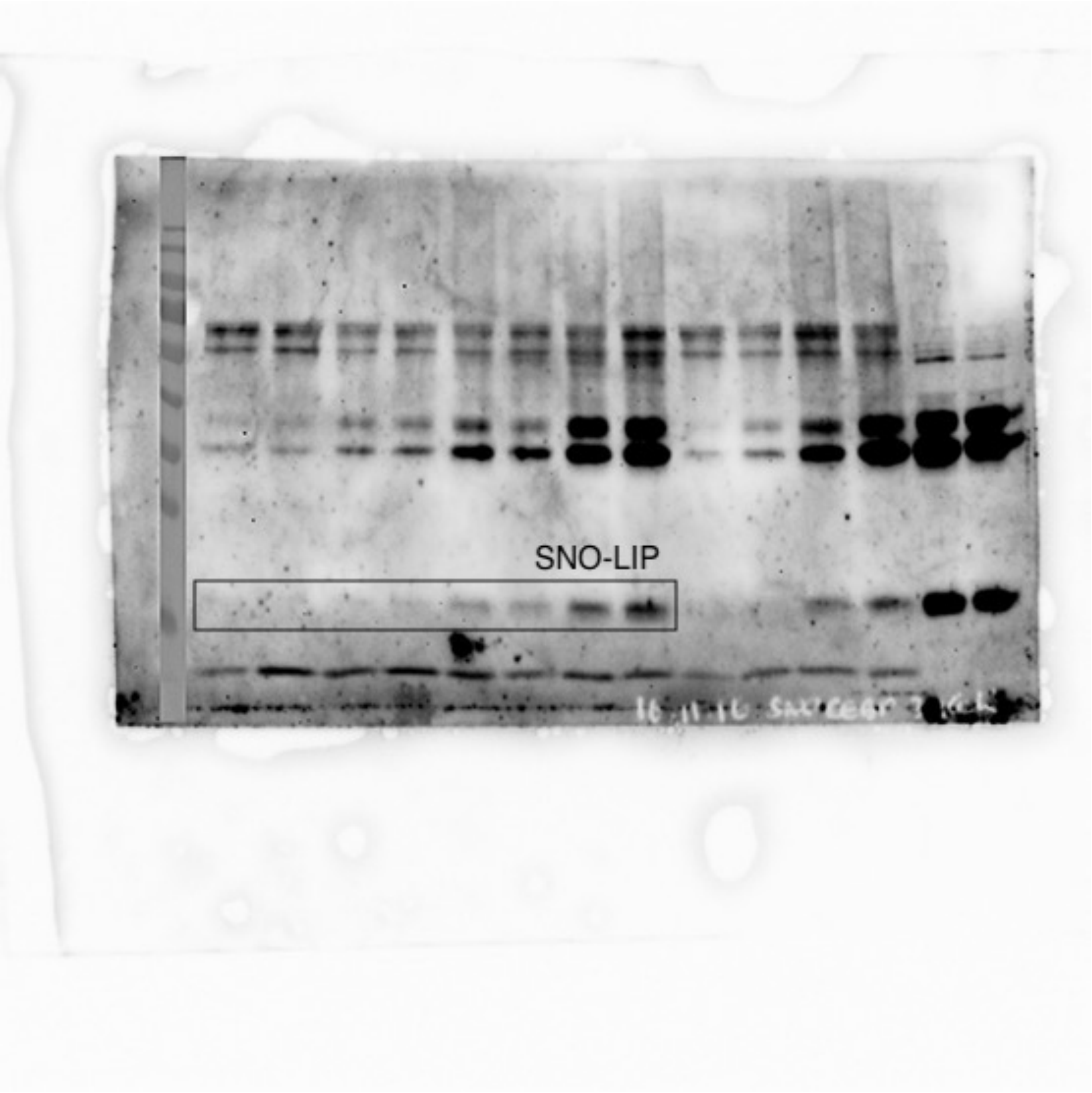

Fig. 8A_SNO-C/EBPβ_LIP

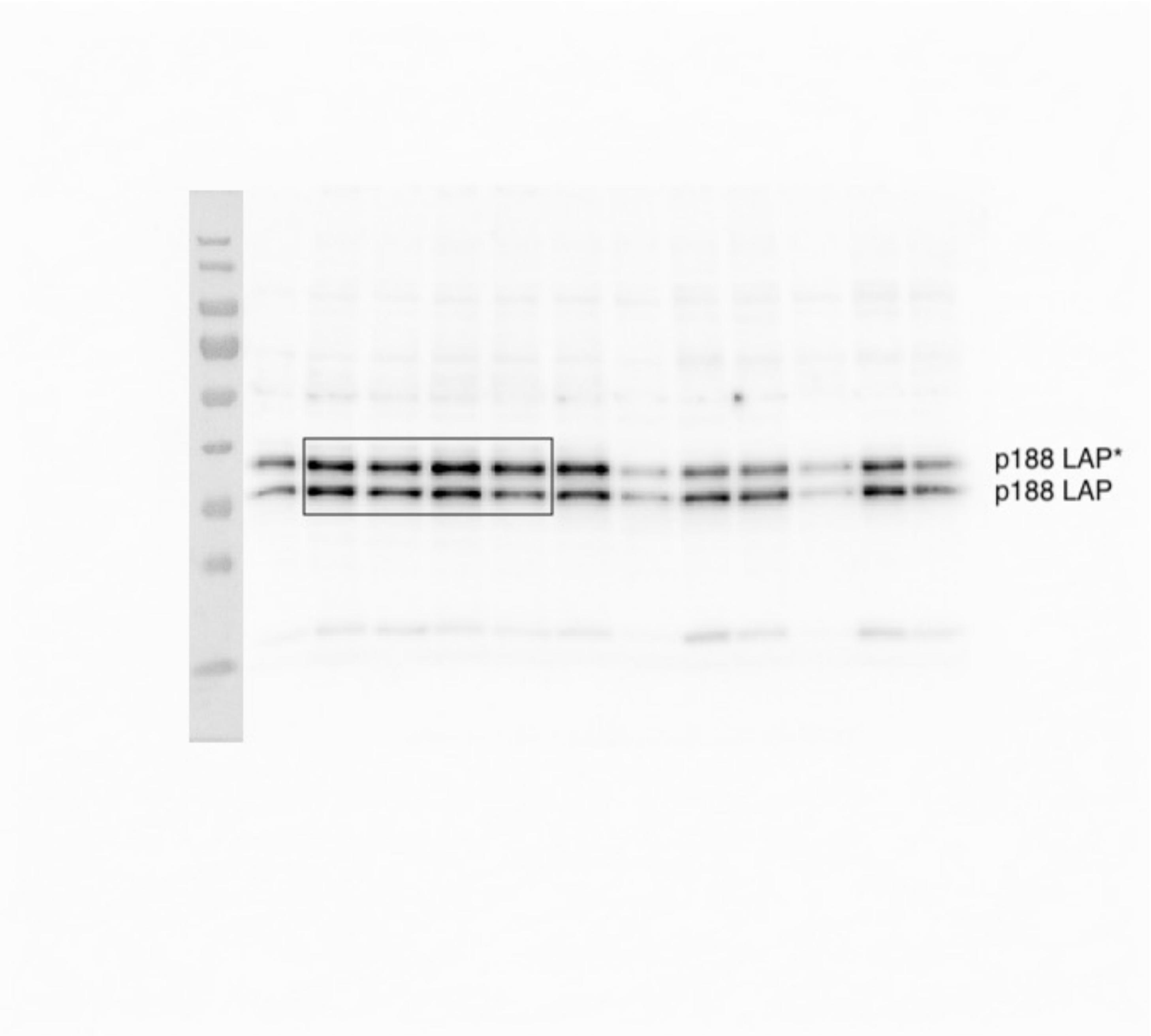

Fig. 8B_p188-LAP* &LAP

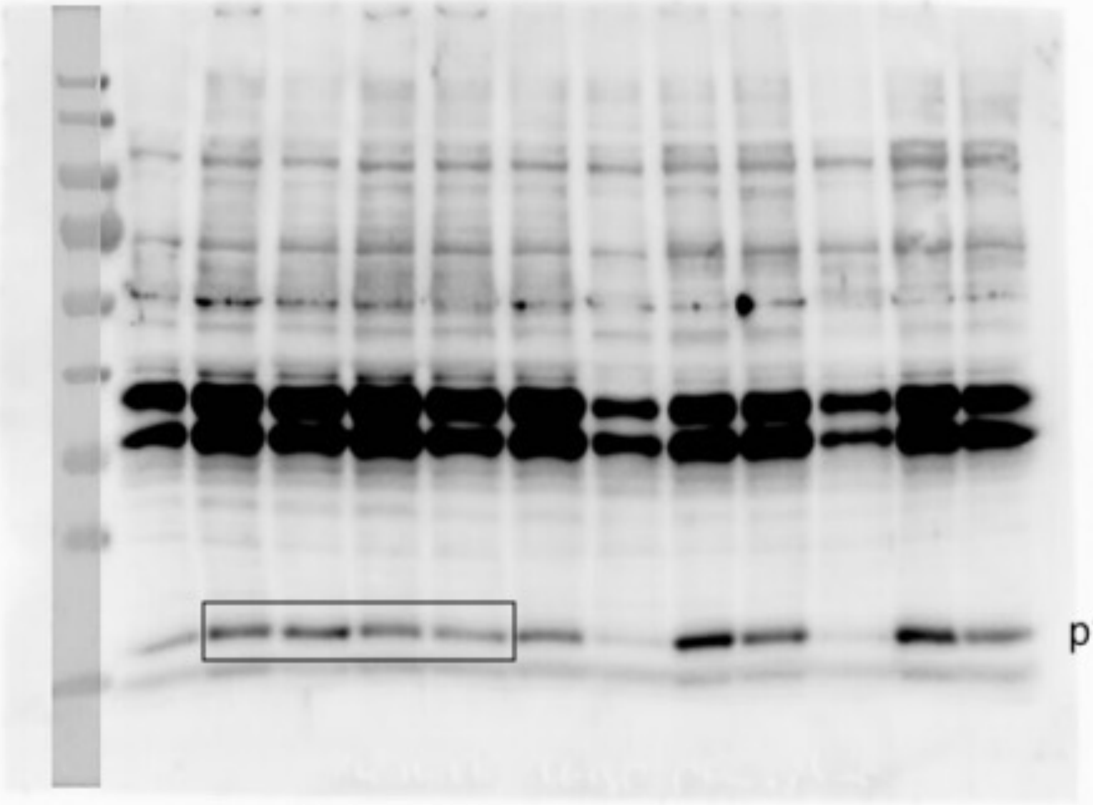

Fig. 8B_p188-LIP

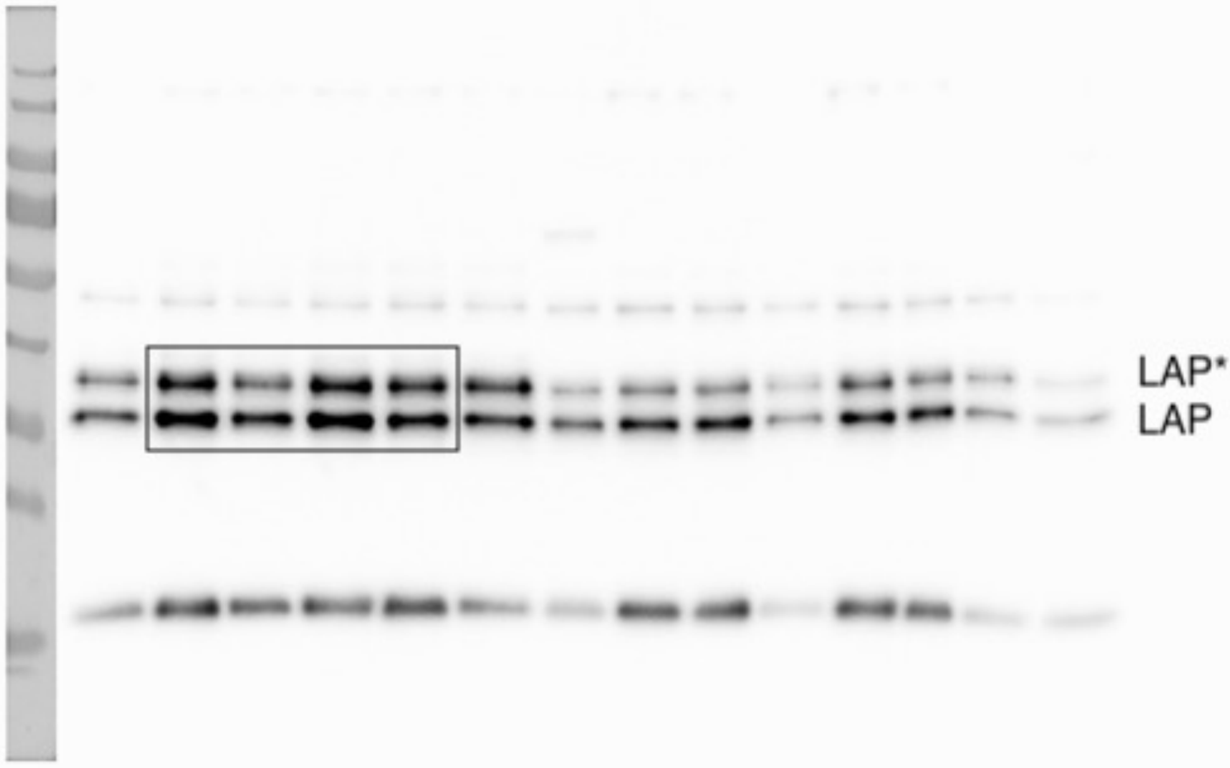

Fig. 8C_ C/EBPβ_LAP* &LAP

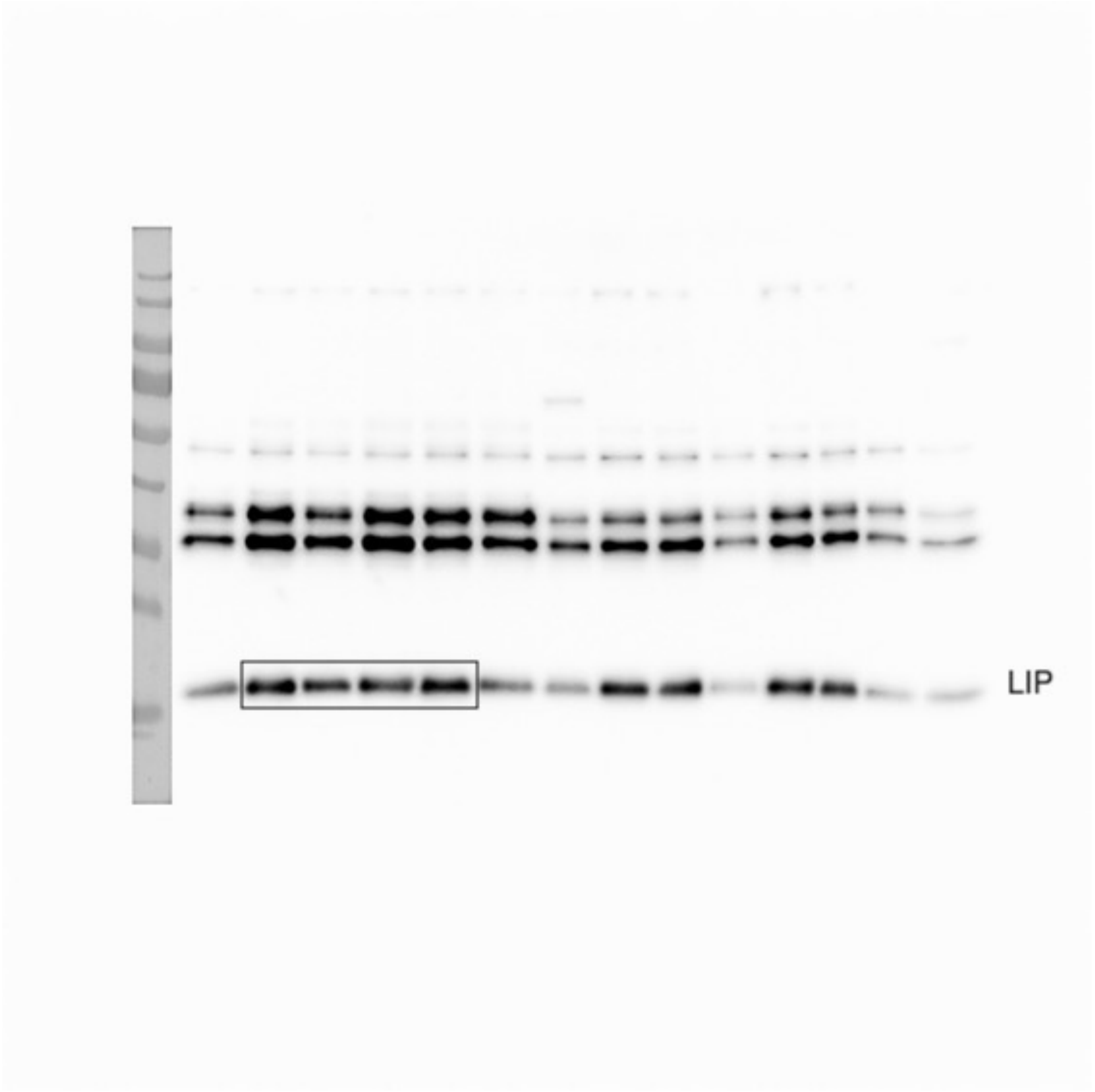

Fig. 8C_ C/EBPβ_LIP

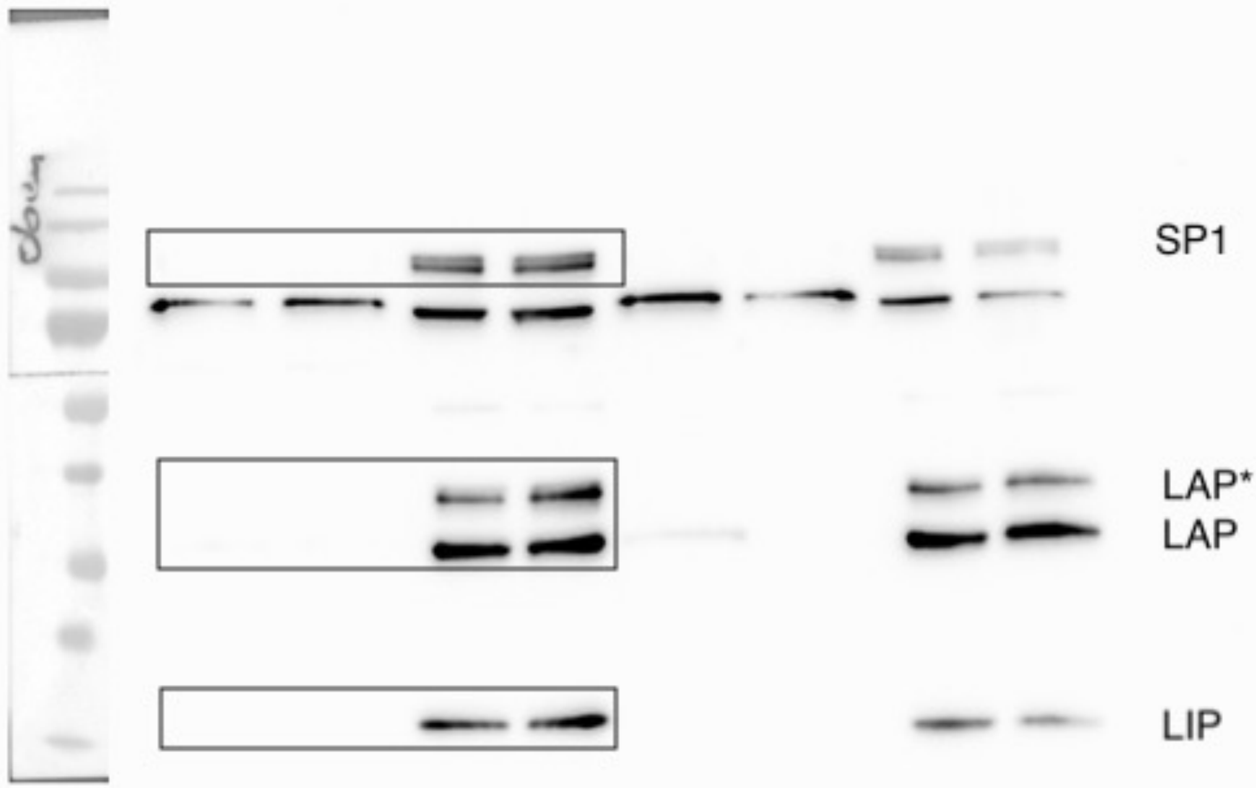

Fig. 8D_ C/EBPβ_LAP*, LAP, LIP, Sp-1

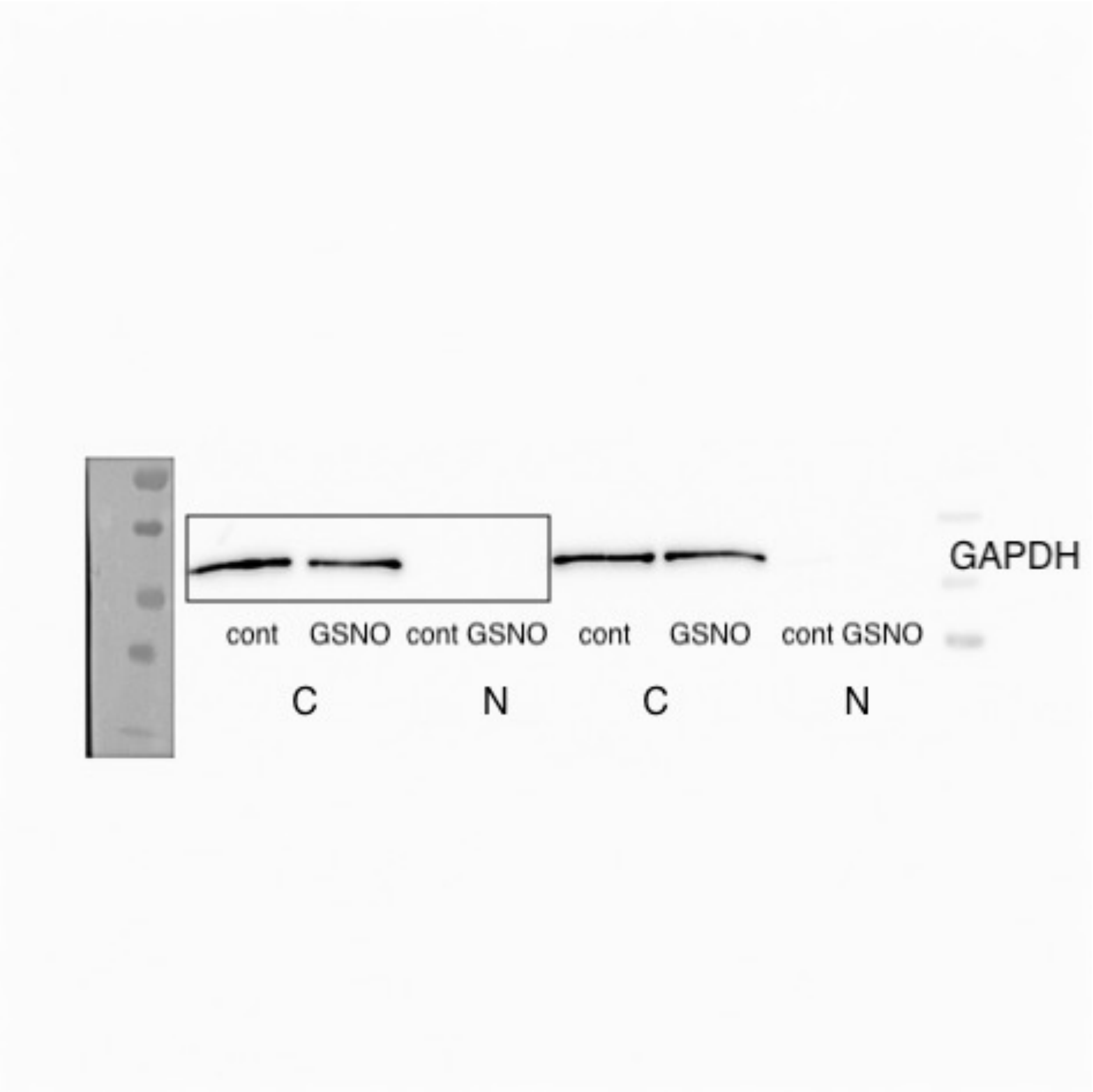

Fig. 8D_GAPDH

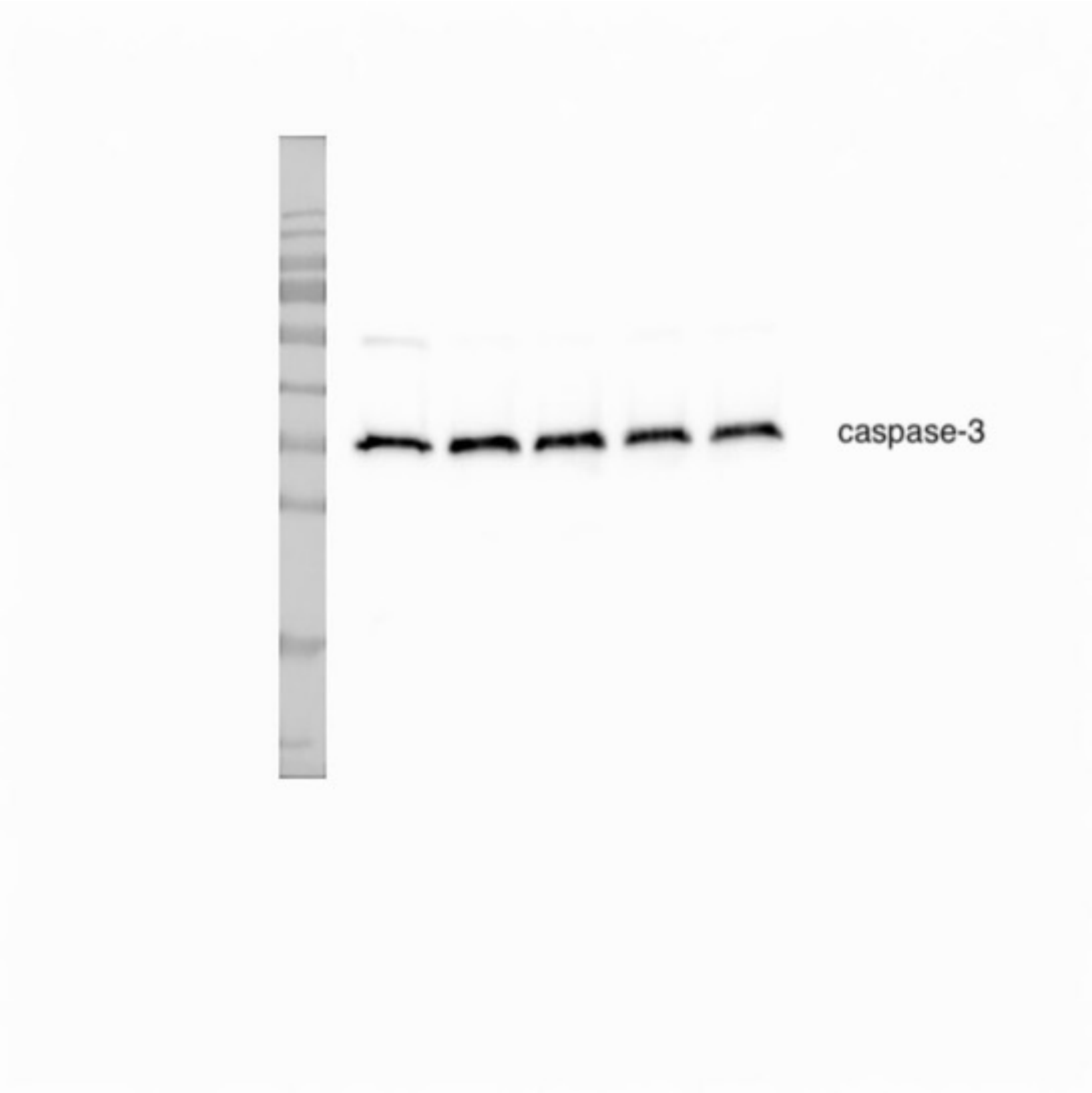

Fig. S1B_caspase

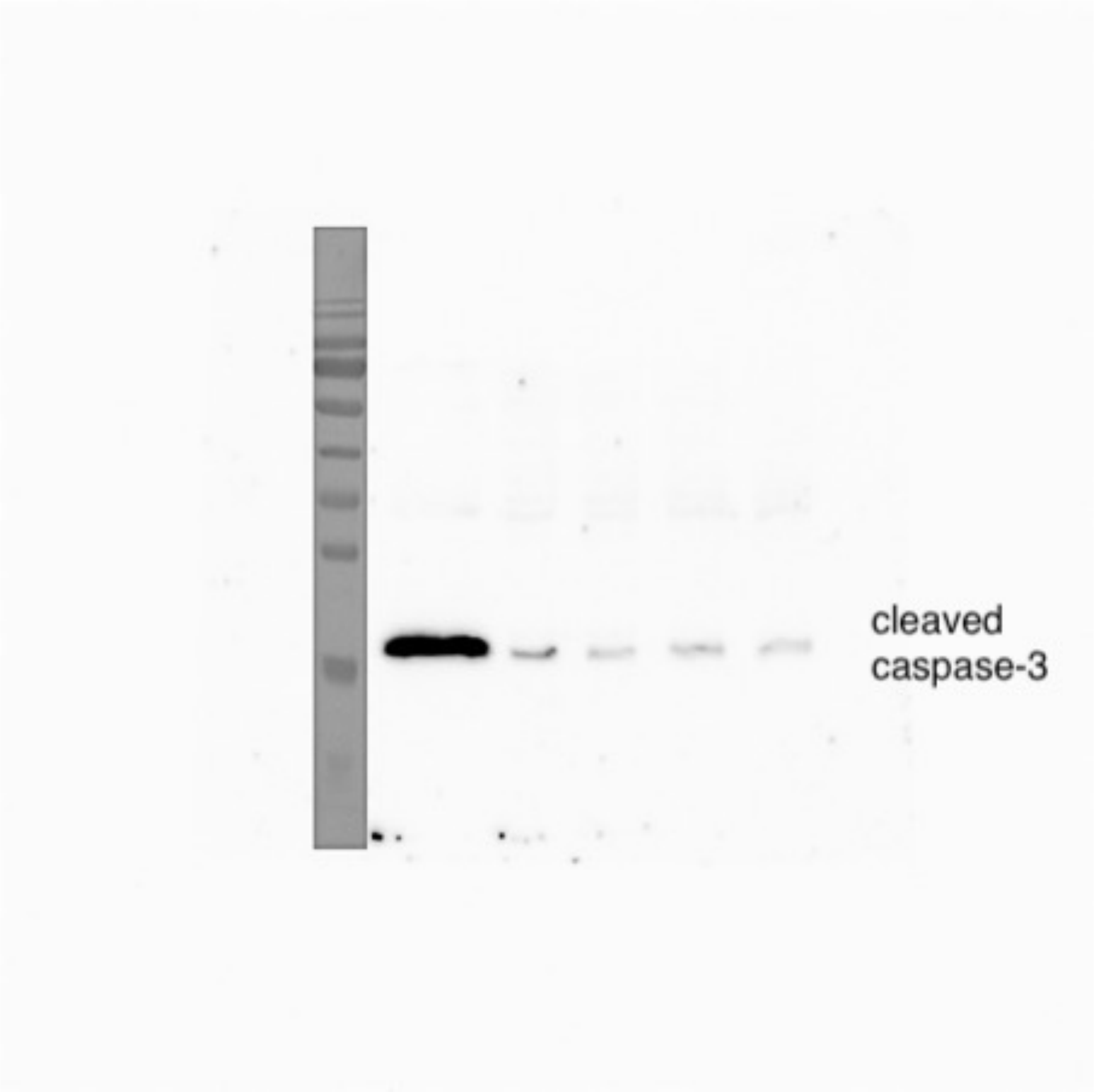

Fig. S1B_cleaved caspase

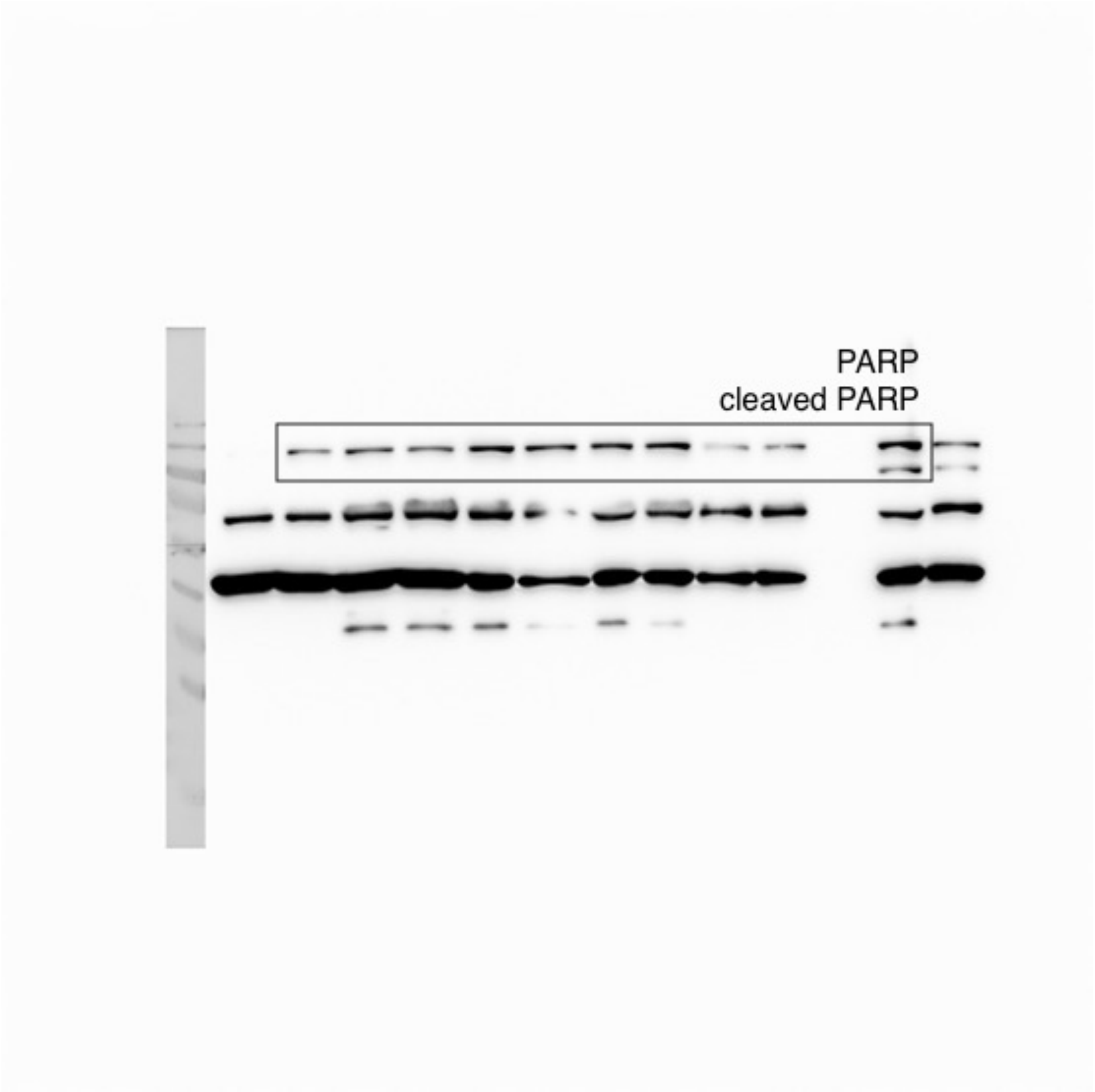

Fig. S1C_PARP/cleaved PARP

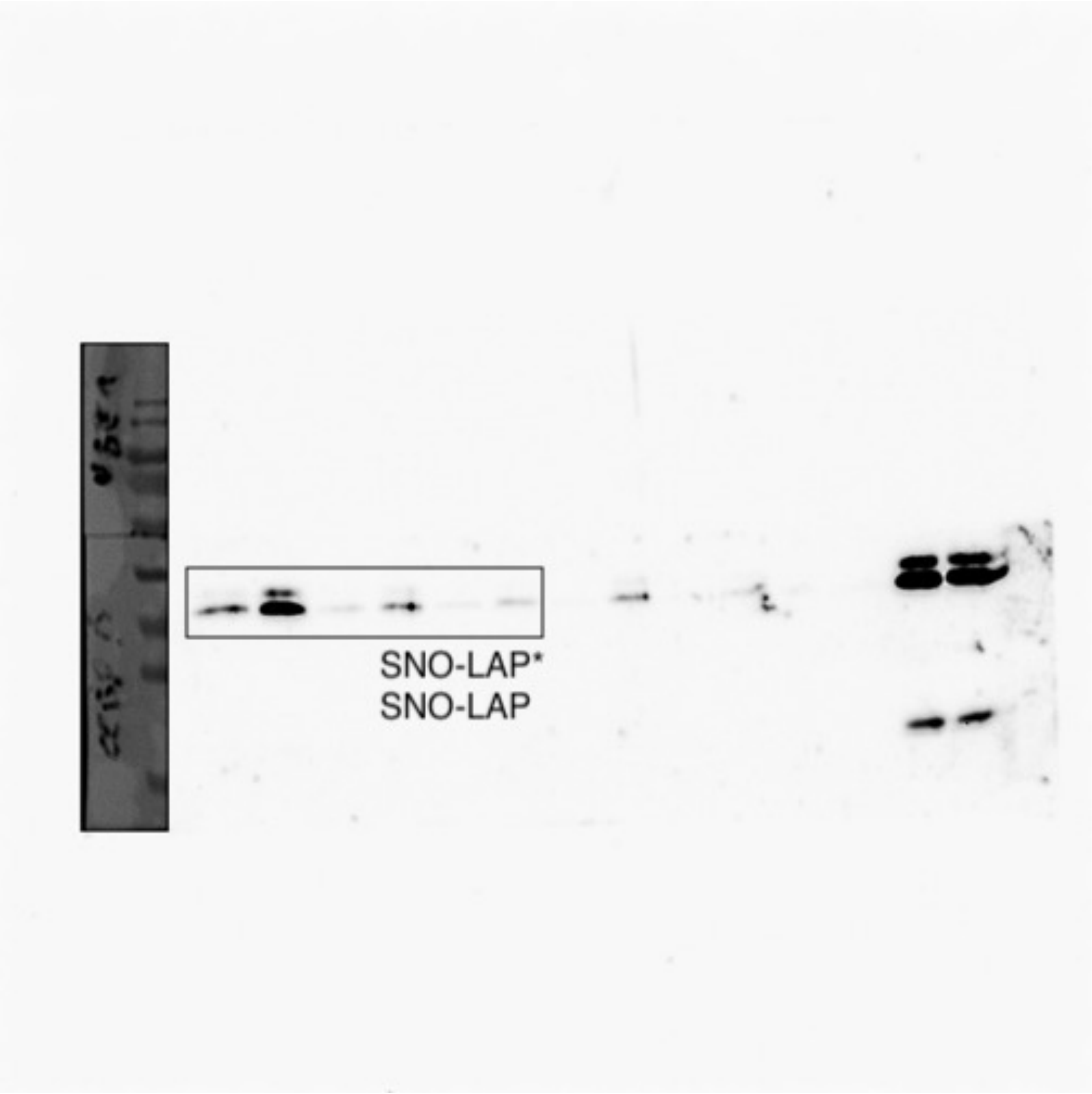

Fig. S2C_SNO-C/EBPβ

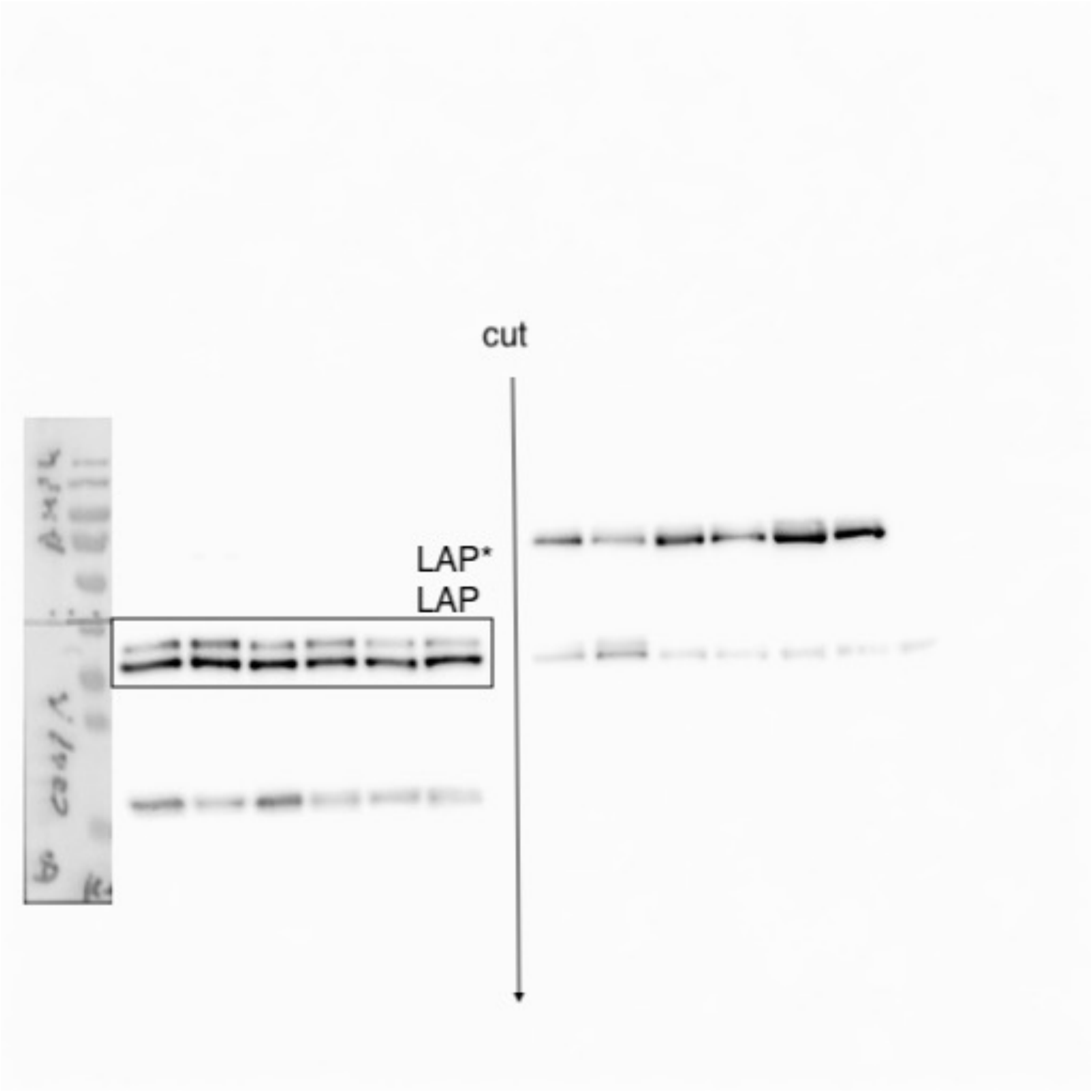

Fig. S2C_ C/EBPβ

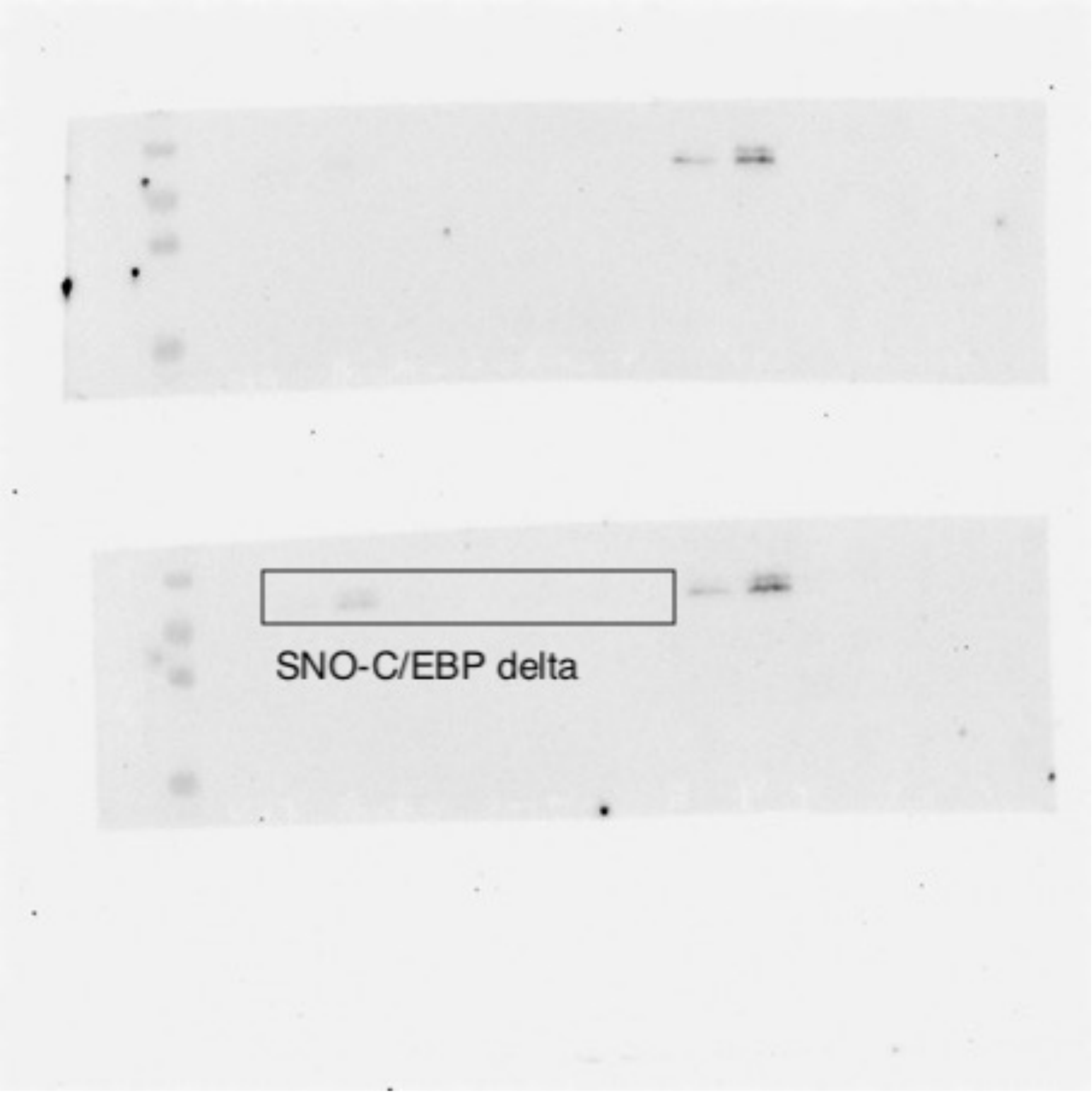

Fig. S2F_SNO-C/EBPδ

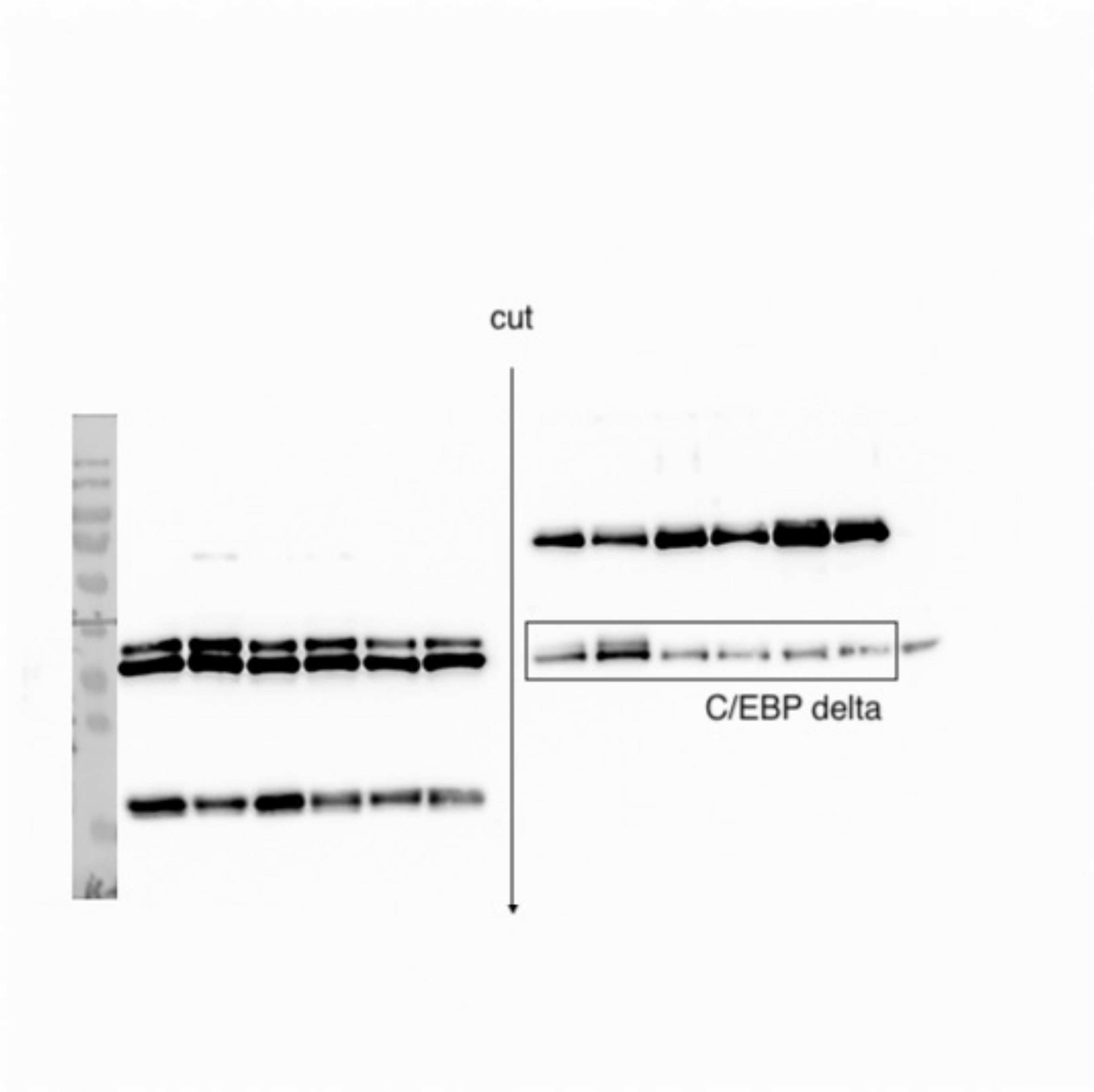

Fig. S2F_ C/EBPδ

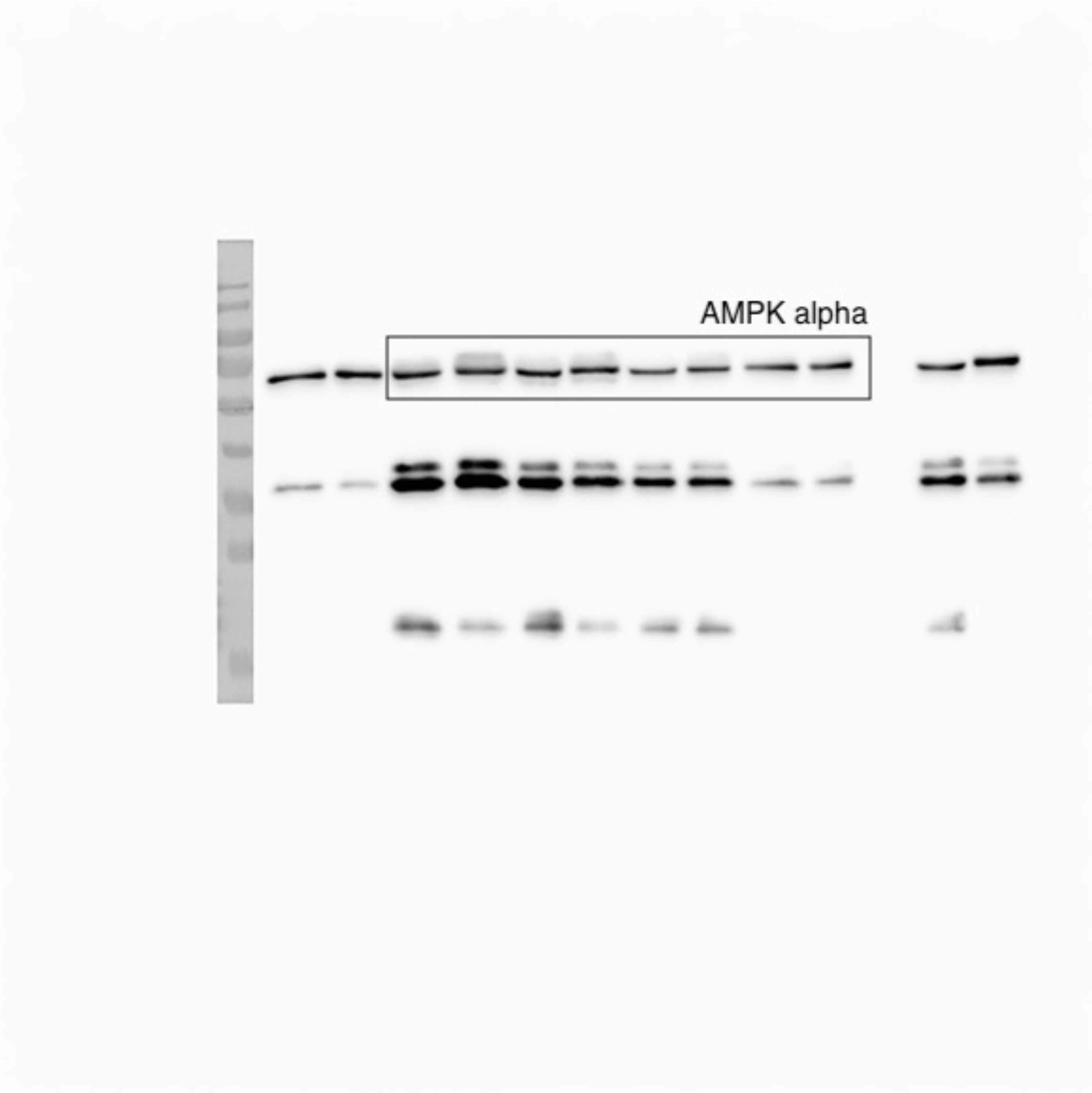

Fig. S3C_AMPKα

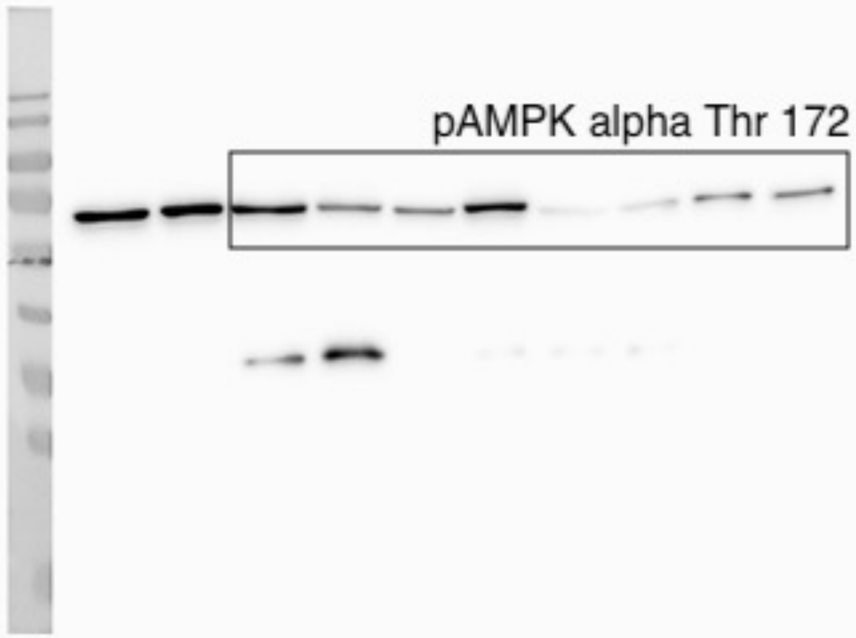

Fig. S3D_p-Thr172-AMPKα
