# Supplementary material for: Author Correction: S-nitrosoglutathione inhibits adipogenesis in 3T3-L1 preadipocytes by S-nitrosation of CCAAT/enhancer-binding protein β
Source: Sci Rep. 2020 Jun 12;10:9846. doi: 10.1038/s41598-020-67063-w (PMC7289844; doi:10.1038/s41598-020-67063-w)
Supplement: Supplementary file 1 — Supplementary Information. [file 41598_2020_67063_MOESM1_ESM.pdf]

SUPPLEMENTAL INFORMATION

S-nitrosoglutathione inhibits adipogenesis in 3T3-L1 preadipocytes by S-nitrosation of CCAAT/enhancer-binding protein β

Marion Mussbacher[1,2], Heike Stessel[1], Teresa Pirker[1], Antonius C.F. Gorren[1], Bernd Mayer[1], and Astrid Schrammel[1*]

[1] Department of Pharmacology and Toxicology, University of Graz, Humboldtstrasse 46, A-8010 Graz, Austria

[2] Center for Physiology and Pharmacology, Department of Vascular Biology and Thrombosis Research, Medical University of Vienna, Schwarzspanierstraβe 17, A-1090 Vienna, Austria

* Corresponding author

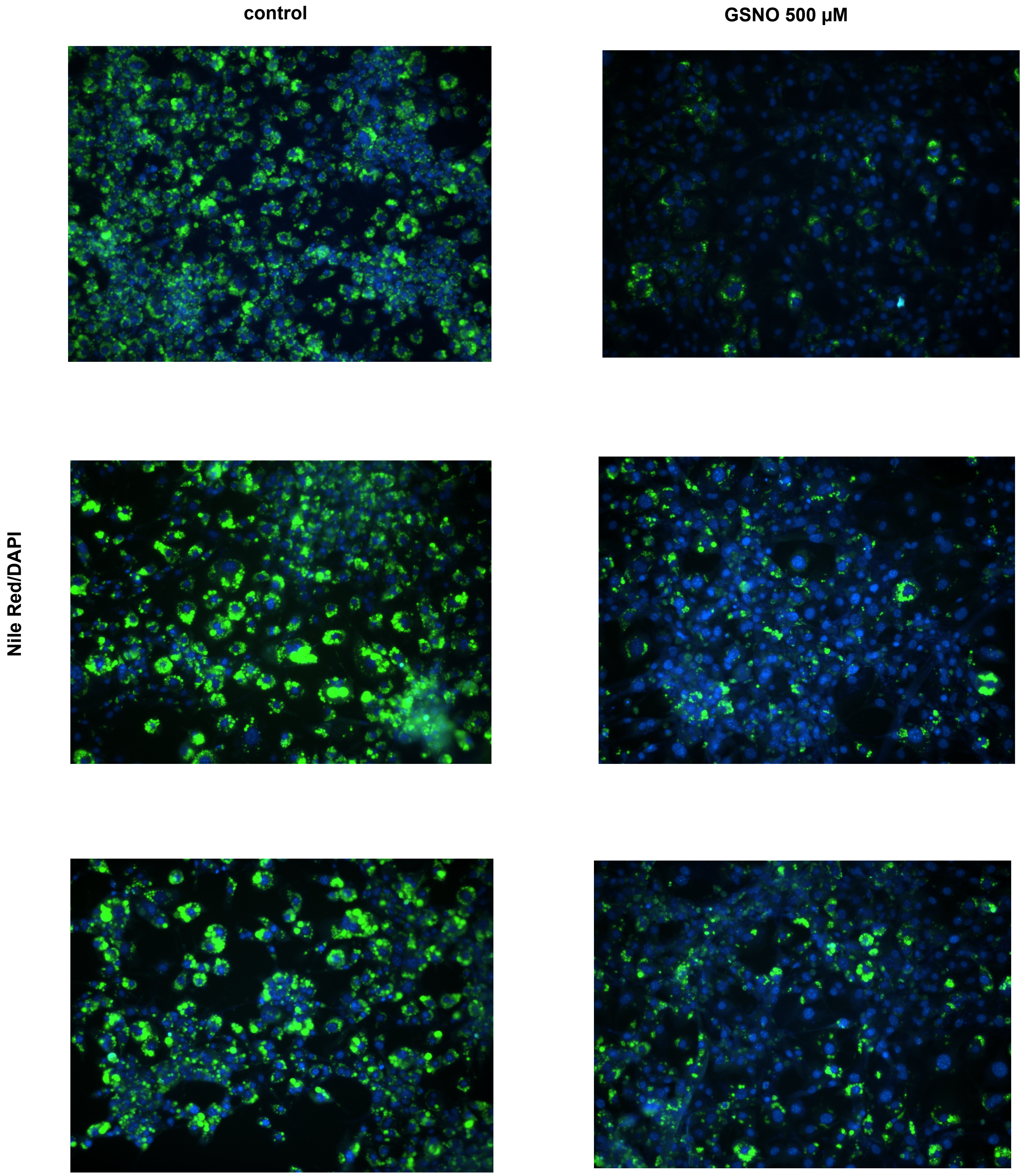

images shown in the manuscript

Fig_2A

control

DETA/NO 100 µM

Nile Red/DAPI

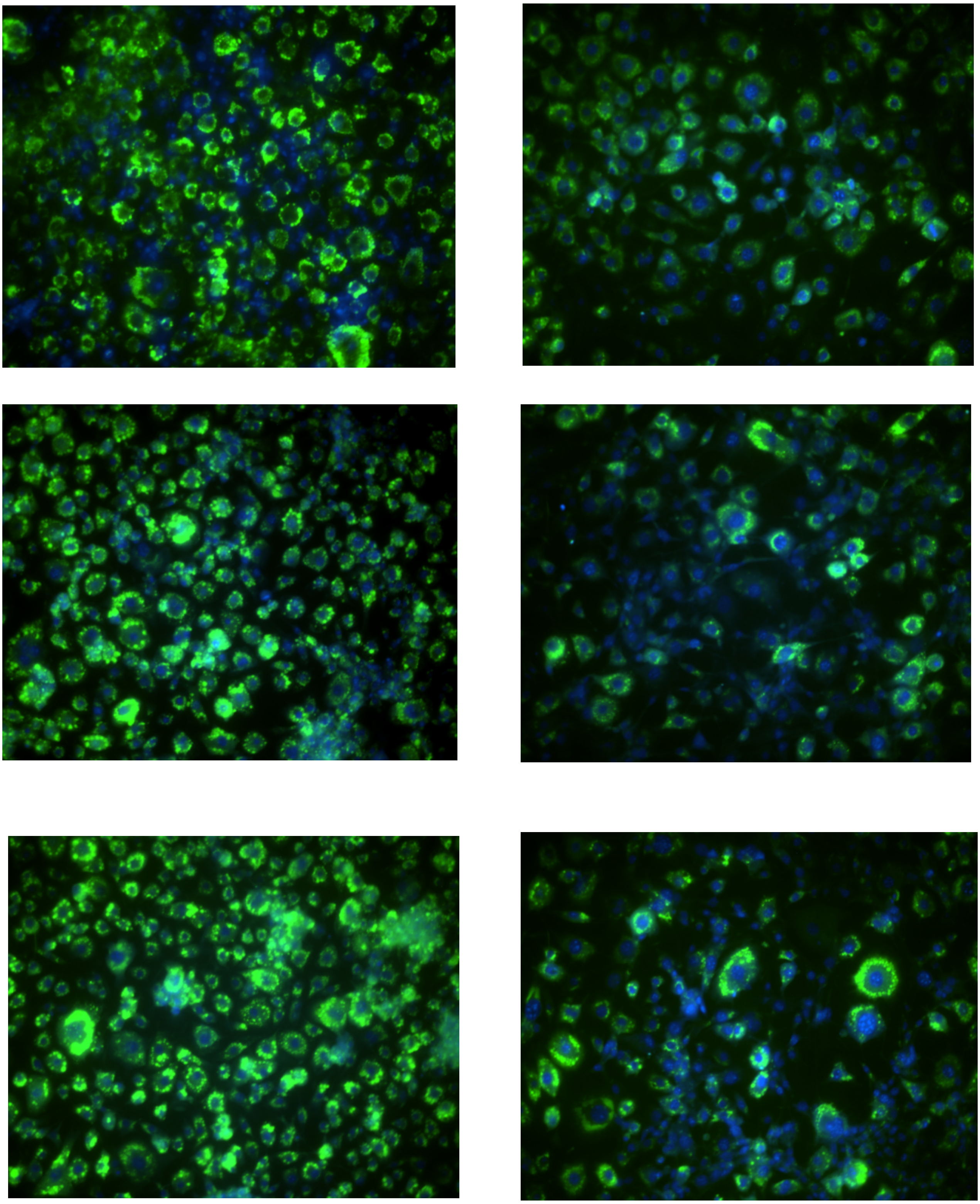

images shown in the manuscript

Fig_3F
